# Cell-type-specific proximity labeling of organ secretomes reveals energy balance-dependent proteomic remodeling

**DOI:** 10.1016/j.celrep.2026.117507

**Published:** 2026-06-12

**Authors:** Kaja Plucińska, Charlotte R. Wayne, Henry Sanford, Boby Mathew, Nathalie Ropek, Stephanie M. Adaniya, Corey Model, Nicolás Gómez-Banoy, Ksenia Morozova, Xiongwen Cao, Jeffrey M. Friedman, Ken H. Loh, Paul Cohen, Ekaterina V. Vinogradova

**Affiliations:** 1 Laboratory of Molecular Metabolism, The Rockefeller University, 1230 York Avenue, New York, NY 10065, USA; 2 Laboratory of Chemical Immunology and Proteomics, The Rockefeller University, 1230 York Avenue, New York, NY 10065, USA; 3 Department of Comparative Medicine, Yale University, School of Medicine, New Haven, CT 06520, USA; 4 Division of Endocrinology, Department of Medicine, Memorial Sloan Kettering Cancer Center, New York, NY 10065, USA; 5 Division of Endocrinology, Diabetes and Metabolism, Department of Medicine, Weill Cornell Medicine, New York, NY 10065, USA; 6 Laboratory of Molecular Genetics, The Rockefeller University, 1230 York Avenue, New York, NY 10065, USA; 7 Present address: Department of Biochemistry, University of Washington, Seattle, WA 98195, USA; 8 Present address: Department of Chemistry, Stanford University, Stanford, CA 94305, USA; 9 Present address: Shanghai Key Laboratory of Regulatory Biology, Institute of Biomedical Sciences, School of Life Sciences, East China Normal University, Shanghai 200241, China; 10 These authors contributed equally; 11 Lead contact

## Abstract

Intercellular communication is critical for maintaining organismal metabolic homeostasis. Here, we develop a method enabling temporally controlled, cell-type-specific labeling of secreted and membrane proteins in key metabolic tissues. The method employs a genetically encoded proximity-labeling strategy by targeting a Cre-dependent TurboID ligase to the endoplasmic reticulum (ER) in ES cell-derived mice. The expression of TurboID in hepatocytes, adipocytes, and B lymphocytes enabled the characterization of cell type-specific ER proteomes at baseline and in response to fasting, inflammation, and dietary obesity, revealing tissue- and perturbation-specific changes and augmenting our understanding of how the proteomes of individual tissues change to regulate systemic energy balance. This comprehensive resource represents an important advance toward understanding both how cell-to-cell communication changes in response to energy balance and how it contributes to these alterations. This method is broadly applicable and provides a means for identifying biomarkers and therapeutic targets across a wide range of tissues.

## In brief

Plucińska et al. developed a genetically encoded TurboID^KDEL^ mouse model that enables cell-type-specific tagging of secreted proteomes *in vivo*. This approach was used to uncover dynamic, state-dependent changes in ER proteostasis and the circulating proteome in response to fasting, inflammation, and obesity, identifying candidate circulating biomarkers of human disease.

## Graphical Abstract

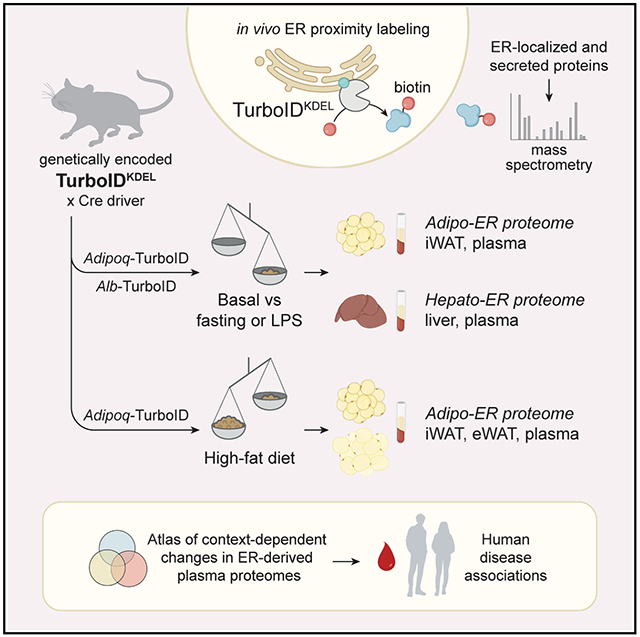

## INTRODUCTION

In multicellular organisms, the maintenance of energy homeostasis requires the orchestration of metabolic processes across and between numerous cell types and tissues. This complex coordination is mediated by secreted factors that can act locally or at a distance to regulate processes, including switching between anabolic and catabolic states and linking nutritional status with other vital functions such as immunity. Autocrine and paracrine factors modulate tissue development and remodeling, while endocrine mediators such as insulin, glucagon,^1^ and leptin^2^ are pivotal for regulating systemic glucose homeostasis, food intake, and energy balance.^3–5^ Moreover, temporal differences in their levels (i.e., acute vs. chronic changes) have distinct effects within and outside cells, including alterations of metabolic flux, signal transduction, endoplasmic reticulum (ER) stress, and energy balance, among many others.

Despite extensive studies, the full extent of the molecular mechanisms that coordinate systemic responses across positive and negative energy balance remains incomplete. During negative energy balance, induced by fasting or systemic inflammation, endocrine and paracrine networks reprogram metabolism to sustain ATP production and preserve glucose for essential tissues, but we still lack an understanding of the complete basis by which organ-specific secretomes regulate metabolism and immune function.^6,7^ Conversely, positive energy balance driven by chronic nutrient excess activates insulin and mTOR signaling to promote anabolic metabolism and lipid storage, but also drives ER stress, mitochondrial dysfunction, and inflammation. How these pathways interact across tissues from adaptive to maladaptive states also remains unresolved. We hypothesized that systematic mapping of tissue- and cell type-specific secretomes in multiple key tissues across energy states would reveal known and novel molecular mediators that respond to and regulate metabolic and immune signaling.

The creation of such an organ-specific atlas of secreted proteins across metabolic states has been a long-standing goal with the potential to provide insights into both normal physiology and disease mechanisms. However, this has been limited by significant technical challenges. First, circulating proteins exhibit a dynamic range of ten orders of magnitude in concentration, making it particularly difficult to identify low-abundance proteins (i.e., in the ng/mL range or lower), which is the level at which most hormones circulate.^8^ Additionally, tracing the source of a secreted protein in blood or other body fluids is not possible with traditional mass spectrometry (MS) because it does not provide information on cell type specificity, nor does it provide insight into paracrine versus endocrine factors. While inferences can be gained from transcriptomic profiling, RNA levels do not always predict secreted protein levels, as they do not account for post-transcriptional and post-translational regulation or secretory dynamics.^9^ Furthermore, the use of *in vitro* models to identify secreted proteins is limited as they do not fully recapitulate *in vivo* biology and are limited to cell types that can be cultured with fidelity.

The use of bioorthogonal amino acid-tagging (BONCAT)^10^ or proximity labeling (PL)^11^ has substantially enhanced our ability to map cell-type selective proteomes by providing precise information on the origin, identity, and spatiotemporal dynamics of intracellular and secreted proteins.^12^ PL catalyzes the proximity-dependent modification of proteins, which can be achieved by the introduction of engineered versions of biotin ligase BirA, such as R118G BirA (BioID) and BirA*G3,^13,14^ TyroID,^15^ and TurboID,^11,16^ each of which promiscuously biotinylates resident or transiently associated proteins in a subcellular compartment in a cell type-specific manner. Further specificity can be achieved by targeting the enzyme to a particular cell type using conditional genetic systems. The biotinylated proteome can then be enriched with streptavidin (SA) beads and identified via MS.^11,12,16^ This was elegantly demonstrated in Drosophila using a muscle-specific ER-anchored BirA*G3 to label classically secreted proteins in response to exercise^14^ and in mice via AAV-mediated expression of an ER-linked BioID^17^ or TurboID fused with Ces61b (ER membrane)^18^ or KDEL (ER lumen). The use of these AAVs has enabled the characterization of secreted proteomes from various cell types, including hepatocytes, myocytes, pericytes, adipocytes, and myeloid cells under different physiologic states *in vivo*.^19,20^ However, the use of AAV can be limited because of the typical artifacts from incomplete or off-target viral transduction and the inflammatory response provoked by viral infection. These limitations can be overcome by the introduction of Cre-dependent expression of TurboID targeted to the secretory pathway *in vivo*. We thus set out to generate a mouse line that could be used to profile secreted proteins in a cell type-specific manner.

Here we report the development and validation of a genetically encoded PL TurboID^KDEL^ mouse for cell type-selective labeling of ER-resident or secreted proteomes *in vivo*. We used this approach to create an atlas of ER-resident and secreted proteomic signatures in two different types of white adipocytes, hepatocytes, and B cells at baseline and in hepatocytes and adipocytes after fasting, inflammation, and early and advanced diet-induced obesity (DIO). We selected these cells for profiling secreted proteomes due to their key roles in interorgan crosstalk as well as anabolic and catabolic processes during altered energy states. Validation of the method allowed us to generate comprehensive, high-resolution data characterizing the levels of ER-localized and secreted proteins, providing new and potentially critical insights into the molecular networks governing energy balance. This included the identification of previously unreported cell-type-enriched circulating mediators linked to changes in energy balance with potential relevance to human health and disease.

## RESULTS

### Cell-type-specific ER-proximity labeling

**Figure 1.**
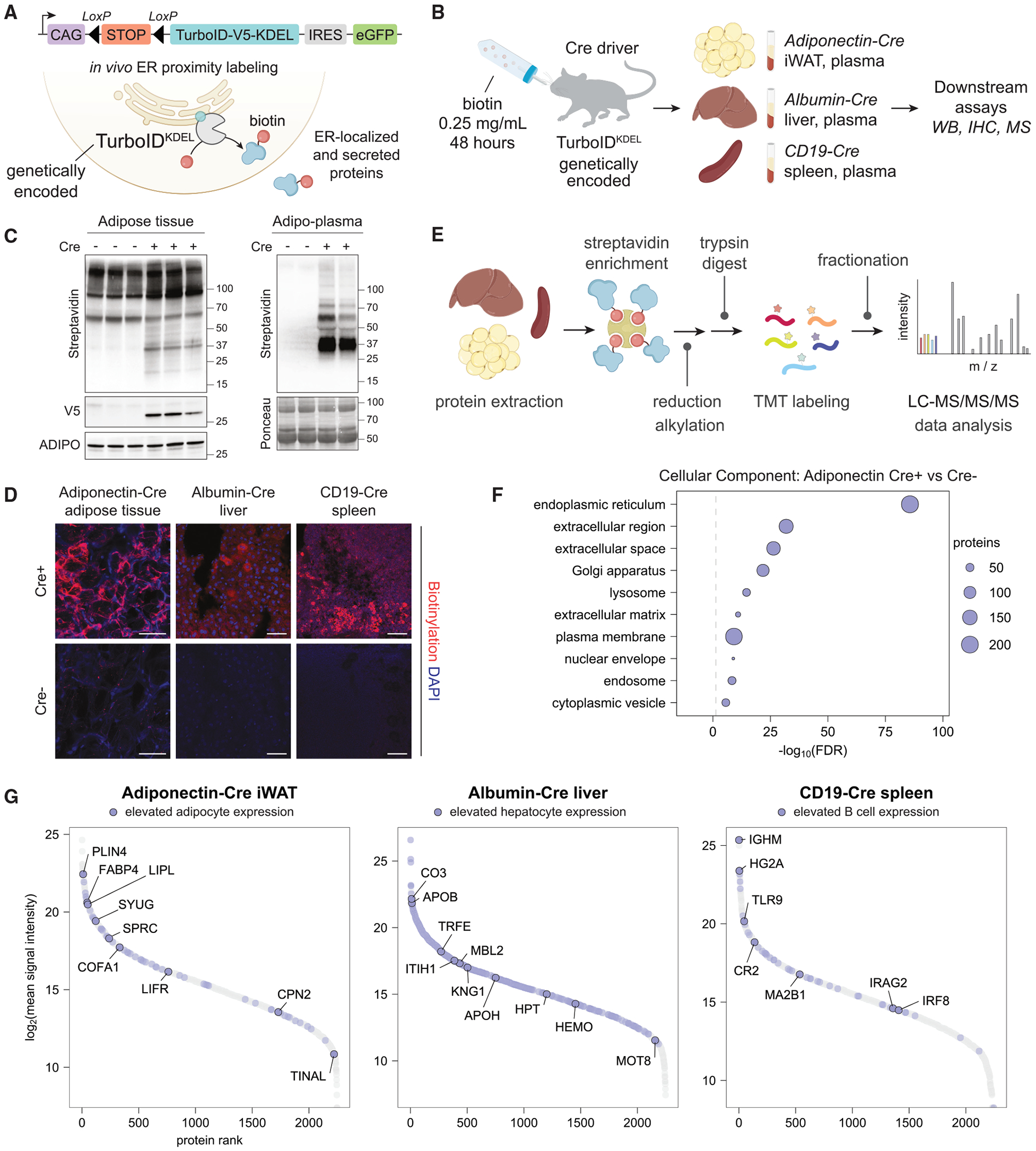
Introduction to TurboID workflow and validation of tissue-specific *in vivo* proximity labeling (A) Schematic of LoxP-flanked TurboID-KDEL construct and ER-localized TurboID proximity labeling system. (B) Experimental design for comparison of three tissues under basal conditions. (C) Western blot shows TurboID-catalyzed biotinylation of inguinal adipose tissue and plasma from biotin-treated Adipoq-TurboID^KDEL^ mice, as well as V5 tag indicative of TurboID expression. (D) Immunohistochemistry images of inguinal white adipose tissue (iWAT), liver, and spleen tissue in TurboID^KDEL^ mice with and without the expression of Adiponectin-Cre, albumin-Cre, or CD19-Cre. Biotinylation is shown in red (streptavidin for iWAT, neutravidin for liver and spleen). Scale bars, 50 μm. (E) Workflow for tissue processing, affinity purification, and analysis by Tandem-Mass-Tag (TMT) mass spectrometry. (F) Pathway analysis illustrates predicted subcellular localization of proteins enriched in basal Adipoq-TurboID^KDEL^ Cre+ relative to Cre− samples. Dashed line indicates the false discovery rate (FDR) of 5%. Gene Ontology term enrichment analysis was performed with GO slim cellular component with the full mouse genome used as the background list. (G) Protein abundance plots highlight cell-type-specific markers expressed in inguinal adipocytes (iWAT), hepatocytes, and splenic B lymphocytes, respectively.

To enable *in vivo* cell-type-specific PL of the secretory pathway proteome in a non-immunogenic system, we developed a knock-in mouse line in which TurboID fused to an ER retention signal (KDEL) and a V5 epitope tag was targeted to the Rosa26 locus (Figure 1A). An upstream LoxP-flanked STOP cassette restricts expression to Cre-expressing cells, enabling cell-specific expression. We crossed this TurboID^KDEL^ line to mice expressing Cre recombinase under the control of cell type-specific promoters, including Adiponectin-Cre,^21^ Albumin-Cre,^22^ and CD19-Cre^23^ for selective expression in adipocytes, hepatocytes, and B lymphocytes (Figure 1B). Biotin supplementation in drinking water (Figure 1B) led to robust biotinylation in tissues and plasma (Figure 1C; Figures S1A–S1C), while intraperitoneal injection produced weaker and less uniform labeling (Figure S1D). The specificity of TurboID expression was confirmed by SA-HRP and V5-tag western blotting (Figure 1C; Figures S1A–S1C) and SA immunohistochemistry (Figure 1D). Cre-negative controls showed only background signal likely reflecting endogenously biotinylated proteins, such as biotin-dependent carboxylases.^11^ The distribution of biotin-labeled proteins was consistent with cell-type-specific TurboID expression, though with a variable degree of biotinylation signal from other tissues not expressing Cre/V5 (i.e., minimal across organs except kidney, inguinal white adipose tissue (iWAT), and liver), likely demonstrating renal clearance of biotin and possibly enhanced tissue crosstalk (Figures S1A–S1C). These experiments also confirmed that TurboID ligase is not secreted into the bloodstream (see V5 expression in all three mouse models).

We performed quantitative bottom-up proteomic analysis of labeled proteins from individual tissues and plasma using Tandem Mass Tag-based multiplexing^24^ (TurboID-TMT). Biotinylated proteins were enriched with SA beads from iWAT, liver, and spleen lysates (Figure 1E), followed by reduction, alkylation, and trypsin digestion. After TMT labeling (Figure S1E), samples were combined, fractionated to increase proteome coverage, and analyzed by MS. Cre-negative controls were used to exclude nonspecific and endogenously biotinylated proteins. Receiver operating characteristic (ROC)-based analysis was applied as previously described^11^ to define specific thresholds identifying ER-localized proteins with fidelity (Figures S1F and S1G; see Supplementary Methods for details). As expected, proteins enriched in Cre+ samples showed Gene Ontology (GO) associations with subcellular localization to the secretory pathway (Figures 1F and S1H; ER, extracellular, and Golgi). Further, many of the proteins were predicted by SignalP^25^ to contain signal peptides (Figures S1H and S1I). Finally, the secretory proteomes from adipocytes, hepatocytes, and B cells were clearly separated by principal component analysis (PCA) (Figure S1J) and included numerous cell-type-enriched proteins annotated in the Human Protein Atlas (Figures 1G; Table S1). Together, these studies validated the use of the genetically encoded TurboID^KDEL^ mouse strain combined with our proteomic pipeline as a robust and versatile platform for characterizing the secreted proteome *in vivo*, as well as ER-resident and ER-transitory proteins such as glycoproteins and membrane receptors.

### *In vivo* models for profiling secretory pathway proteomes under negative energy balance

**Figure 2.**
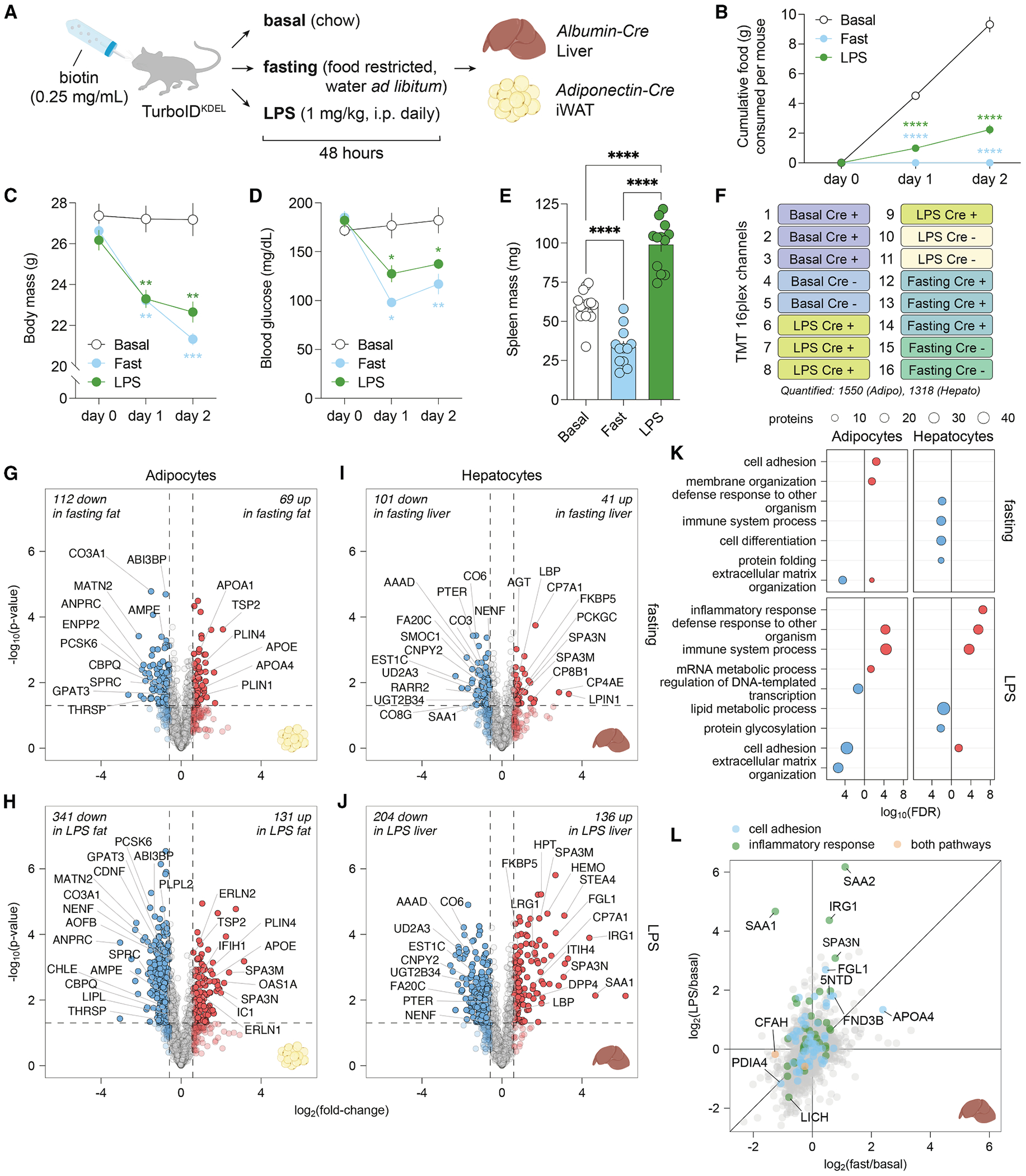
Proteomic response of hepatocytes and adipocytes to negative energy balance (A) Experimental design to induce negative energy balance in mice, including 48 h of LPS treatment or fasting in two tissue-specific driver lines, adiponectin-Cre (adipocytes) and albumin-Cre (hepatocytes). (B) Cumulative food consumption in LPS and fasting conditions over 48 h. LPS and fasting were each compared to the basal condition by two-way ANOVA with Tukey’s multiple comparisons test (****, *p* < 0.0001). Error bars show mean ± SEM; n = 6–8 cages per condition. Mice include Cre+, Cre−, and wild-type genotypes. (C) Body mass change over 48 h of fasting or LPS. Conditions compared to basal by two-way ANOVA with Tukey’s multiple comparisons test (ns, *p* > 0.05, not shown; **, *p* < 0.01; ***, *p* < 0.001). Error bars show mean ± SEM; n = 6–8 mice per condition. Mice include Cre+, Cre−, and wild-type genotypes. (D) Change in blood glucose over 48 h of fasting or LPS. Conditions compared to basal by two-way ANOVA with Tukey’s multiple comparisons test (ns, *p* > 0.05, not shown; *, *p* < 0.05; **, *p* < 0.01). Error bars show mean ± SEM; n = 6–8 mice per condition, including Cre+, Cre−, and wild-type genotypes. (E) Spleen mass following 48 h of fasting or LPS. Conditions compared by one-way ANOVA with Tukey’s multiple comparisons test (****, *p* < 0.0001). Error bars show mean ± SEM; *n* ≥ 12 mice per condition. (F) Representative TMT channel assignment for a 16-plex LC-MS/MS/MS experiment compares three conditions (basal, LPS, and fasting) in mice with and without a cell-type-specific Cre driver. The number of proteins passing the ROC cutoff is also shown. (G and H) Volcano plots show log_2_(fold-changes) in protein expression in inguinal white adipocyte (iWAT) samples from fasted or LPS-treated Adipoq-TurboID^KDEL^ mice relative to basal. Dashed lines represent cutoffs of *p* value <0.05 and FC > 1.5. (I and J) Volcano plots show log_2_(fold-changes) in protein expression in hepatocytes from fasted or LPS-treated Albumin-TurboID^KDEL^ mice relative to basal. Dashed lines represent cutoffs of *p* value <0.05 and fold-change >1.5. (K) Pathway analysis of protein expression changes. Dot plots show GO terms with <0.05 FDR when using Fisher’s exact test against an experiment-specific background list. (L) Scatterplot shows protein expression changes in hepatocytes from fasted (x axis) or LPS-treated (y axis) albumin-TurboID^KDEL^ mice. Select pathways are labeled based on Gene Ontology annotations.

We next used this approach to characterize the secretory proteome in adipocytes and hepatocytes *in vivo* under two conditions of negative energy balance: 1) a 48-h fast and 2) lipopolysaccharide (LPS) treatment (1 mg/kg, intraperitoneally once daily for 48 h) to induce inflammation-associated anorexia.^7^ The 48-h fasting was administered to induce a well-characterized systemic metabolic shift that enables clear detection of fasting-dependent proteomic changes and pathways associated with this drastic nutrient deprivation. CD19-TurboID mice were not used in metabolic experiments due to poor plasma proteome labeling at baseline. Both perturbations were compared to *ad libitum*-fed, saline-injected controls. Mice were concurrently provided with biotin-supplemented drinking water (0.25 mg/mL) for 2 days, enabling TurboID-mediated biotinylation *in vivo* (Figure 2A). Tissues (liver and adipose) and plasma were collected from animals between zeitgeber time (ZT) ZT5-6, during the light phase, to minimize variation associated with circadian rhythms.

Cumulative food intake was significantly reduced in LPS-treated mice compared to controls (Figure 2B). Both interventions led to significant decreases in body mass and blood glucose (Figures 2C and 2D). While both sets of conditions are associated with negative energy balance, we also found that LPS induced splenomegaly, indicative of selective activation of immune responses, which was not observed in fasted mice (Figure 2E).

Following each perturbation, biotinylated proteins were enriched from iWAT and liver from Adipoq-TurboID^KDEL^ and Albumin-TurboID^KDEL^ mice, respectively, and quantified by TurboID-TMT proteomics (Figures 2F; Tables S1 and S2). MS identified 2,460 proteins in iWAT lysates, of which 1,550 passed the ROC-based enrichment threshold. Liver samples were processed similarly in a separate TurboID-TMT 16-plex experiment, with 2,608 proteins identified in hepatocyte-ER, of which 1,318 met the ROC-based enrichment threshold. As expected, many enriched proteins contained predicted signal peptides by SignalP (376 in iWAT and 343 in liver; Table S2).

### Fasting and LPS trigger distinct remodeling of the secretory proteome in adipocytes

Fasting had highly significant effects on the white adipocyte proteome, with 181 differentially expressed proteins (*p* < 0.05; fold change, FC > 1.5) (Figure 2G). Fasting reduced the abundance of ECM components, including ABI3BP (−0.8 log_2_FC), MATN2 (−2.1 log_2_FC), SPRC (−1.2 log_2_FC), and CO3A1 (−1.5 log_2_FC), suggesting that it induced extensive extracellular matrix (ECM) remodeling. Concomitantly, proteins involved in lipid synthesis and storage (GPAT3, −0.6 log_2_FC; THRSP, 1.5 log_2_FC) and adipose tissue expansion (ENPP2, −2.0 log_2_FC) were downregulated. In contrast, fasting upregulated lipid metabolism and transport proteins, including apolipoproteins (APOE, 0.7 log_2_FC; APOA1, 0.9 log_2_FC; APOA4, 1.2 log_2_FC) and lipid droplet assembly proteins such as perilipins (PLIN1, 1.2 log_2_FC; PLIN4, 0.9 log_2_FC), consistent with lipid mobilization from adipose stores. Adipocyte secretome data were also enriched in proteins involved in lipolysis, such as PNPLA2,^26^ LIPE,^27^ and ABHD5.^28^ In addition, several proteases and aminopeptidases (including CBPQ, −0.7 log_2_FC; AMPE, −0.8 log_2_FC; and PCSK6, −0.8 log_2_FC) were downregulated. While fasting is known to induce profound metabolic reprogramming in adipose tissue,^29^ the reduced expression of these enzymes may suggest diminished peptide processing and proteolytic activity under nutrient deprivation.

Acute LPS treatment induced even more extensive changes of the adipocyte secretory proteome (Figure 2H), with 341 proteins significantly downregulated and 131 upregulated (*p* < 0.05; FC > 1.5). Consistent with the negative energy balance observed in both conditions, LPS-treated adipocytes also showed downregulation of proteins involved in lipid metabolism, including LIPL (−0.6 log_2_FC), PLPL2 (−0.6 log_2_FC), GPAT3 (−0.8 log_2_FC), and THRSP (−1.0 log_2_FC), reduced expression of ECM components,^30^ and upregulation of the adipogenesis inhibitor TSP2 (0.6 log_2_FC).

LPS also induced several markers of inflammation in adipocyte-ER, including interferon-stimulated genes (IFIH1, 0.9 log_2_FC; OAS1A, 2.2 log_2_FC) and negative regulators of inflammation, such as serine protease inhibitor family members (SPA3C, 1.9 log_2_FC; SPA3M, 2.4 log_2_FC; IC1, 1.8 log_2_FC). We also observed increased ER Lipid Raft-Associated Proteins 1 and 2 (ERLN1 and 2, 1.0 and 0.9 log_2_FC, respectively), suggesting the activation of mechanisms that mitigate ER stress, which potentially results from the marked induction of many proteins.^31,32^ Like fasting, LPS treatment led to the downregulation of proteases, including CBPQ (−0.7 log_2_FC), AMPE (−0.6 log_2_FC), and PCSK6 (−1.5 log_2_FC). However, unlike fasting, LPS also induced a significant decrease in the secretion of neurotrophic factors (CDNF, −1.3 log_2_FC; NENF, −1.1 log_2_FC) and proteins involved in neurotransmitter degradation (AOFB, −0.6 log_2_FC; CHLE, −0.9 log_2_FC).

### Fasting and LPS trigger distinct remodeling of the hepatocyte secretory proteome

Fasting induced the differential expression of 142 hepatocyte secretory pathway proteins (*p* < 0.05; FC > 1.5) (Figure 2I; Table S3). Multiple complement components (CO3, −0.8 log_2_FC; CO6, −1.3 log_2_FC; CO8G, −1.3 log_2_FC) and the acute-phase hepatokine SAA1 (−1.3 log_2_FC) were downregulated in line with the suppression of immune responses during fasting^33,34^ and the central role of hepatocytes in complement activation and acute-phase responses. Proteins involved in lipid catabolism and energy conservation were also decreased, including AAAD (−0.9 log_2_FC), EST1C (−0.7 log_2_FC), and PTER (−0.8 log_2_FC).^35^ Other notable decreases include neurotrophic and growth-related factors (NENF, −0.6 log_2_FC; CNPY2, −0.8 log_2_FC), the glucose-responsive hepatokine SMOC1 (−0.7 log_2_FC), and the secretory pathway kinase FA20C (−0.9 log_2_FC), which regulates ECM remodeling. Consistent with the role of the liver in phase II detoxification via glucuronidation, ER-localized glucuronosyltransferases (including UD2A3, −0.8 log_2_FC; UGT2B34, −0.8 log_2_FC) were suppressed. In contrast, fasting induced proteins involved in gluconeogenesis together with enzymes involved in bile acid biosynthesis and fatty acid metabolism (CP7A1, 1.7 log_2_FC; PCKGC, 1.3 log_2_FC; CP8B1, 1.6 log_2_FC; CP4AE, 2.8 log_2_FC; and LPIN1, 3.3 log_2_FC). Overall, these data were consistent with metabolic reprogramming toward fuel production, which differed from the response in adipocytes (Table S3).

LPS led to broader changes of the hepatocyte secretory pathway proteome compared to fasting (Figure 2J), inducing the differential expression of 340 proteins (*p* < 0.05; FC > 1.5). This included the upregulation of acute-phase proteins (LBP,^36^ SAA1,^37^ HEMO, HP; log_2_FCs of 0.6, 4.7, 2.3, and 2.0, respectively), anti-inflammatory modulators (SPA3M, SPA3C, FGL1; log_2_FCs of 1.9, 3.1, and 2.7, respectively), and integrators of inflammatory and metabolic signaling (DPP4, STEA4; log_2_FCs of 1.6 and 3.1, respectively). We also noted the upregulation of leucine-rich α2-glycoprotein 1 (LRG1, log_2_FC 1.5),^38,39^ a factor implicated in hepatic steatosis and insulin resistance.

### Shared and tissue-specific responses to fasting and LPS in secretory proteomes

To capture shared and distinct responses across cell types and negative energy balance models, we performed GO pathway analysis of “biological processes” enriched in adipocyte and hepatocyte secretory pathway proteomes following fasting or LPS (Figures 2K and 2L; Figures S2A–S2F). As expected, LPS promoted inflammatory and defense response pathways in both cell types (Figures 2K, 2L, and S2A). In contrast, immune-related pathways were downregulated in fasted hepatocytes.^40,41^

Proteins regulating cell adhesion and ECM organization were the most enriched processes in adipocyte-derived secretory proteomes, responding distinctly between fasting and inflammatory stress (Figures 2K and S2B). Key quality control processes mediated in the ER, including protein folding and protein glycosylation, were also significantly enriched in both hepatocytes and adipocytes (Figures 2K; Figures S2C and S2D), while lipid metabolic processes were downregulated in LPS-stimulated hepatocytes (Figures 2K and 2L).

Comparison of hepatocyte and adipocyte secretory pathway proteins highlighted the shared and tissue-specific responses to fasting and LPS (Figures S2F–S2H). LPS induced inflammatory regulators (SPA3C, SPA3M, SAMP) and downregulated lipid anabolic enzymes, including the major lipase EST1D, in both experiments, while many of the acute phase response proteins were only detected in liver samples. We validated these findings by performing western blot analysis of independent wild-type mouse cohorts treated with biotin during fasting or LPS treatment; EST1D and CO6, both downregulated in LPS and fasting conditions in the liver by MS, were consistently reduced by western blotting (Figures S2I and S2J).

### Changes in circulating hepatokines and adipokines in response to negative energy balance

**Figure 3.**
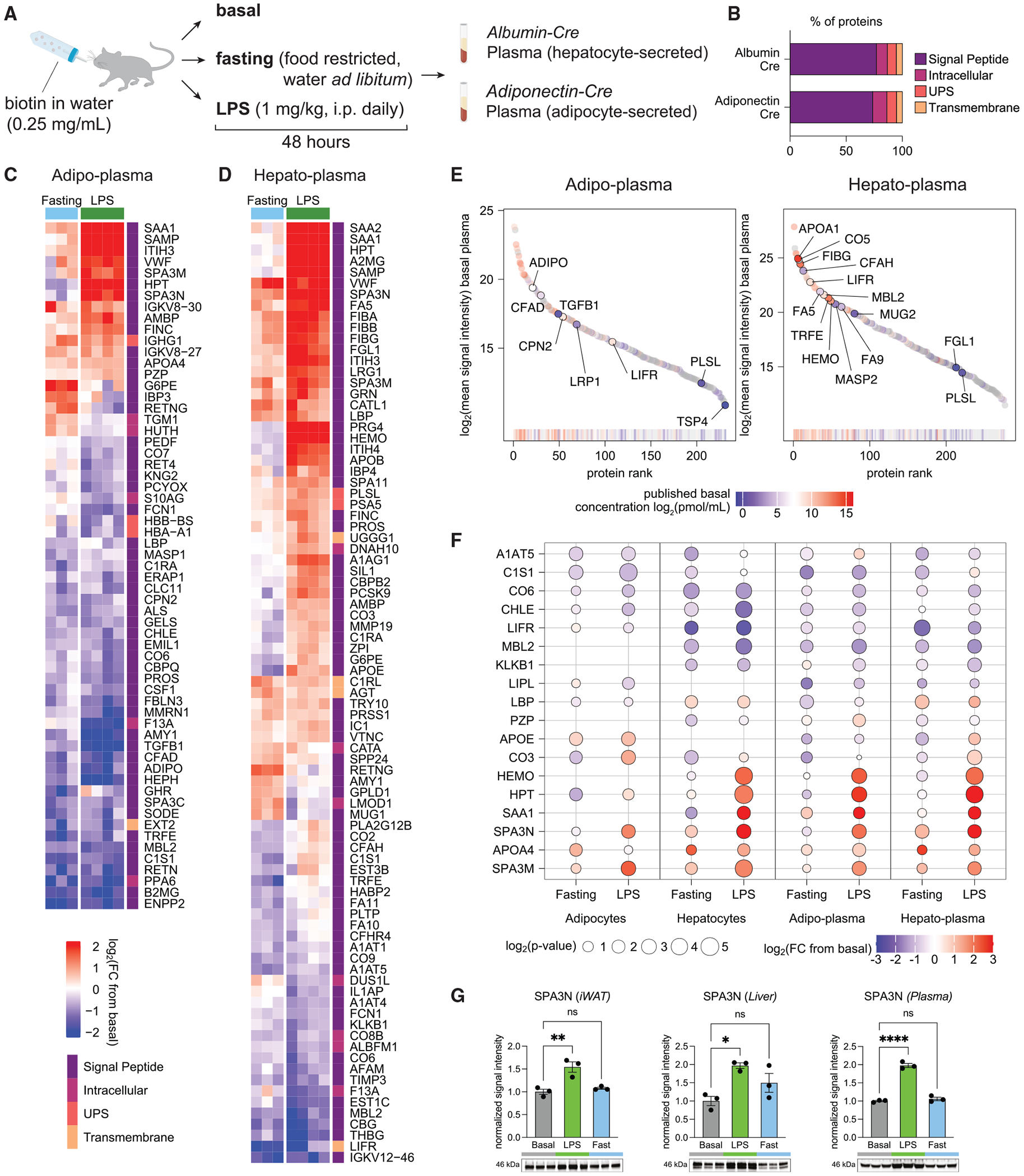
Hepato-plasma and adipo-plasma proteomic responses to negative energy balance (A) Experimental design to capture circulating adipokines and hepatokines in response to fasting or LPS in TurboID mice. (B) Bar plot of proteins quantified in albumin-TurboID^KDEL^ and Adipoq-TurboID^KDEL^, with protein class labeled based on SignalP and OutCyte predictions. (C) Heatmap of differentially expressed proteins in adipo-plasma (*p* < 0.05, FC > 1.5 in either of the negative-energy-balance conditions compared to basal). Proteins are hierarchically clustered based on FC. (D) Heatmap of differentially expressed proteins in hepato-plasma (*p* < 0.05, FC > 1.5 in either of the negative-energy-balance conditions compared to basal). Proteins are hierarchically clustered based on FC. (E) Rank plot of mean signal intensity values of proteins quantified by TurboID-TMT in basal adipo- and hepato-plasma, colored by known plasma concentrations. (F) Dot plot of log_2_FC and *p* values of selected proteins detected in adipocytes, hepatocytes, and plasma across negative-energy-balance conditions. (G) Validation by western blot of SPA3N upregulation in response to LPS in bulk iWAT, liver, and plasma. Western blot band intensity was normalized to the total intensity of the corresponding lane in a stain-free gel image. Comparison performed by one-way ANOVA with Dunnett’s multiple comparisons test (ns, *p* > 0.05; *, *p* < 0.05; **, *p* < 0.01; and ****, *p* < 0.0001). Error bars show mean ± SEM.

Plasma proteins were collected from basal, LPS-injected, and fasted Albumin-TurboID^KDEL^ and Adipoq-TurboID^KDEL^ mice, enriched with SA beads, TMT-labeled, and analyzed by MS to define cell-type-specific circulating secretory proteins (Figures 3A–3D and S3A–S3C), hereafter referred to as the ‘‘hepato-plasma proteome’’ and ‘‘adipo-plasma proteome,’’ respectively. Consistent with selective capture of circulating proteins, plasma datasets were significantly enriched for proteins predicted to have a signal peptide (>70%; Figure 3B; Table S1).

To evaluate the dynamic range of plasma proteins detected, we compared basal plasma reporter ion intensities for 279 proteins that were found to intersect with a previously published atlas of mouse plasma proteome with reported absolute concentrations^42^ (Figures 3E, S3D, and S3E). Proteins with the highest concentrations included apolipoprotein A-I (APOA1, 47.5 nmol/mL) and fibrinogen gamma chain (FIBG, 9.3 nmol/mL), whereas detected proteins with the lowest concentrations were plastin-2 (PLSL, 1.4 pmol/mL), fibrinogen-like protein 1 (FGL1, 0.8 pmol/mL), and thrombospondin 4 (TSP4, 0.7 pmol/mL), suggesting sub-nanomolar sensitivity. Albumin, which was filtered out as a common MS contaminant^43^ in our analysis pipeline, was also one of the top quantified proteins and had a basal concentration of 137.3 nmol/mL. In contrast, several proteins expressed by adipocytes and relatively non-abundant in total plasma, including LRP1 and CFAD (7.8 and 67.2 pmol/mL, respectively), were detected in the adipo-plasma proteome (Figures 3E and S3D), further validating the enrichment strategy for detecting secreted factors from their tissues of origin even at low abundance. Hepatocyte-enriched proteins FA5, FA9, MASP2, and CFAH were similarly overrepresented in the hepato-plasma proteome by TurboID^KDEL^ relative to absolute concentrations from total plasma (Figures 3E and S3E).

A subset of biotinylated proteins we detected in liver and/or iWAT tissues was also found in the plasma-derived samples (Figure 3F; Figures S3F–S3J). The overlap and concordance between tissue and plasma proteomes are particularly informative, as they enable the identification of candidate proteins with potential endocrine, autocrine/paracrine, or dual functions. This integrated approach represents an advance over prior TurboID secretome studies, which focused exclusively on plasma proteomes and therefore could not resolve tissue retention versus systemic distribution. Intersects between tissue and plasma proteomes were higher in the Albumin-TurboID^KDEL^ (Pearson’s R = 0.74 for LPS, R = 0.68 for fasting; Figures S3I and S3J) compared to the Adipoq-TurboID^KDEL^ mice (R = 0.51, R = 0.54; Figures S3G and S3H), likely reflecting differences in protein secretion kinetics and clearance rates between hepatocytes and adipocytes, as well as the potential contribution of other fat depots to the circulating adipo-proteome.

Proteins detected exclusively in adipocytes from tissue may represent factors retained within cells and the local microenvironment, consistent with autocrine or paracrine signaling roles, although differences in MS sensitivity between tissue and plasma cannot be excluded. For example, LGALS1, NID1, and TGM2 have established functions in adipocyte differentiation, adipose tissue morphogenesis, and ECM organization. In contrast, several circulating proteins, including IGF1, CSF1, and MBL2, were identified exclusively in the adipocyte-derived plasma fraction, suggesting potential roles as circulating mediators.

Similarly, analysis of Albumin-TurboID^KDEL^ hepatocyte and plasma proteomes identified well-characterized liver-derived endocrine proteins, such as TTHY, ALS, and AFAM, predominantly in the plasma fraction. Notably, IGF1, a factor with both endocrine and paracrine functions, was detected only in the intracellular proteome. This may reflect limitations in plasma proteomic coverage, where highly abundant proteins such as ALB and IgGs still obscure low-abundance factors.

Both fasting and LPS stimulation suppressed the plasma levels of several complement components, including CO6 and C1S1 (Figure 3F), which are produced in both tissues. Conversely, serine protease inhibitors SPA3C and SPA3M, which target cathepsins and granzymes, were upregulated in adipocytes and hepatocytes, raising the possibility that they serve potential protective functions during inflammatory and metabolic stress (see also Figures S3G and S3J). Likewise, APOA4 induction likely reflects a protective response to negative energy balance, given its reported roles in lipid mobilization and anti-inflammatory signaling.

Several acute-phase and inflammation-responsive proteins, including CO3, HEMO, and HPT, showed divergent regulation between LPS stimulation and fasting, consistent with distinct state-dependent functional roles. In contrast, the acute-phase protein LBP was decreased in both states of negative energy balance in adipocytes and the adipo-plasma proteome but increased in hepatocytes and the hepato-plasma proteome (Figure 3F), indicating cell-specific regulation. Similarly, upon fasting, G6PE, an enzyme in the pentose phosphate pathway, was downregulated in both iWAT and adipo-plasma, but upregulated in hepatocytes and hepato-plasma (Figures S3H and S3I). We also confirmed the upregulation of SPA3N (Figure 3F) in response to LPS by immunoblotting of tissue and plasma samples from wild-type mice (Figure 3G).

Together, the observed correlations between tissue and plasma proteomes, as well as instances of divergence under fasting and LPS stimulation, highlight the complex interplay between post-translational and systemic regulatory mechanisms governing hepatokine and adipokine secretion in response to negative energy balance.

### The white adipocyte tissue secretory proteome in mice fed an HFD for 15 weeks

**Figure 4.**
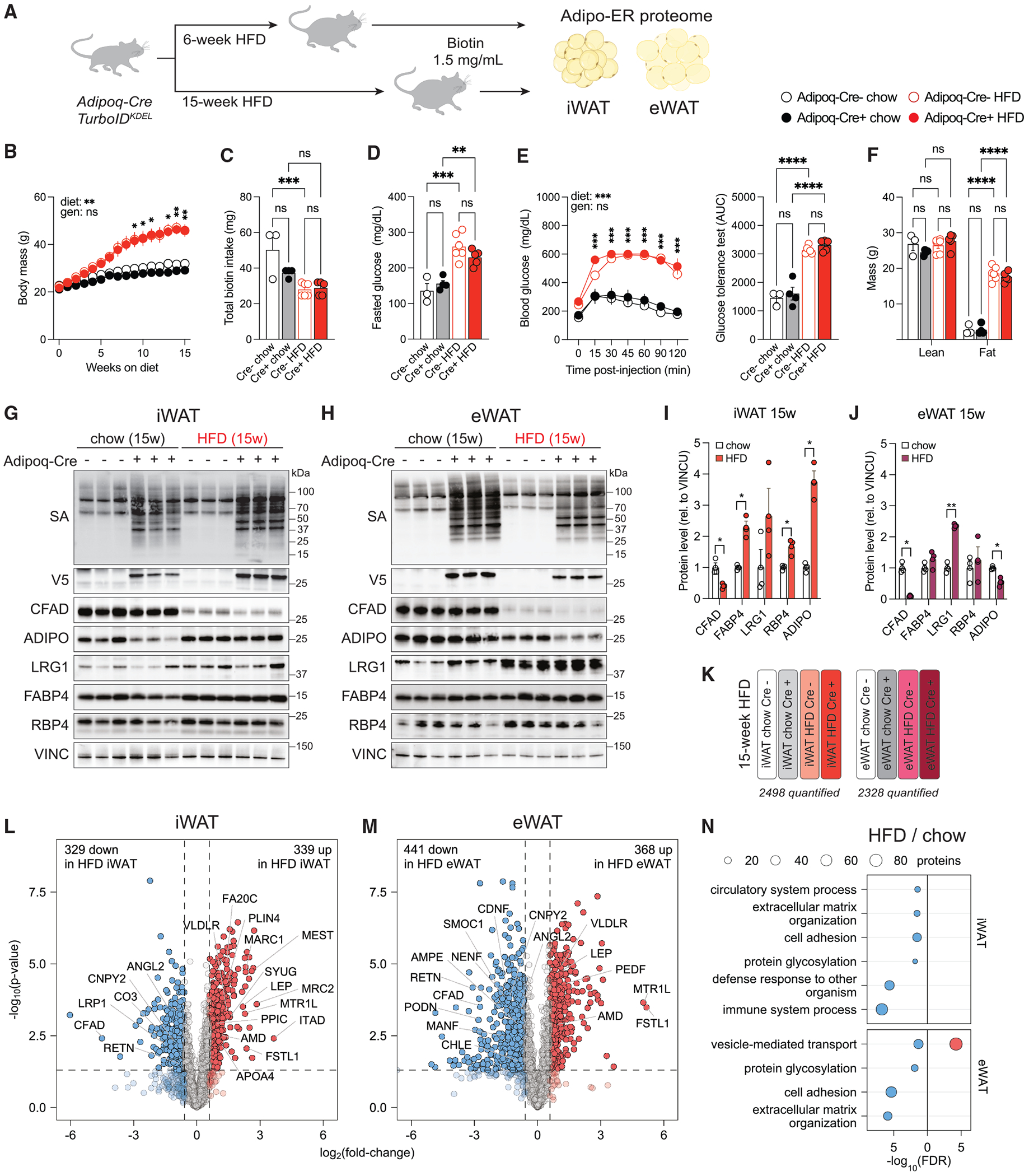
Proteomic responses of inguinal and epididymal adipocytes in Adipoq-TurboID^KDEL^ mice to advanced obesity (A) Experimental design to capture the secretory pathway proteome in inguinal (iWAT) and epididymal white adipocytes (eWAT) of obese Adipoq-TurboID^KDEL^ mice after 6 and 15 weeks of high-fat diet (HFD) feeding. (B) Body weights of mice across 15 weeks of HFD feeding. Biotin (1.5 mg/mL) was administered in drinking water for 7 days on the last week of feeding. Conditions were compared to basal by two-way ANOVA with Tukey’s multiple comparisons test (ns, *p* > 0.05, not shown; *, *p* < 0.05 and **, *p* < 0.01). Error bars show mean ± SEM; n = 4–7 mice per condition. (C) Total biotin intake in mice after 7 days. Conditions were compared to basal by ordinary one-way ANOVA with Šidák multiple comparisons test (ns, *p* > 0.05 and ***, *p* < 0.001). Error bars show mean ± SEM; n = 3–7 mice per condition. (D) Fasted glucose in mice fed chow or HFD for 15 weeks. Conditions were compared by a basal ordinary one-way ANOVA with Šidák multiple comparisons test (ns, *p* > 0.05; **, *p* < 0.01; and ***p < 0.001). Error bars show mean ± SEM; n = 3–7 mice per condition. (E) Glucose tolerance in mice fed chow or HFD for 15 weeks and area under the curve (AUC). Conditions were compared to basal by two-way ANOVA with Tukey’s multiple comparisons test (ns, *p* > 0.05, not shown; ***, *p* < 0.001). Error bars show mean ± SEM; n = 3–7 mice per condition. AUC was compared using ordinary one-way ANOVA with Šidák multiple comparisons test (ns, *p* > 0.05; ****, *p* < 0.0001). Error bars show mean ± SEM; n = 3–7 mice per condition. (F) Fat and lean mass of Adipoq-TurboID^KDEL^ mice after 15 weeks of HFD feeding. Conditions were compared to basal by two-way ANOVA with Tukey’s multiple comparisons test (ns, *p* > 0.05; ****, *p* < 0.0001). Error bars show mean ± SEM; n = 3–7 mice per condition. (G and H) Western blot validation of protein biotinylation using streptavidin-HRP, tissue-specific V5 expression, and levels of known adipokines, including complement factor D (CFAD), adiponectin (ADIPO), leucine-rich glycoprotein 1 (LRG1), fatty acid-binding protein 4 (FABP4), and retinol-binding protein 4 (RBP4), in bulk iWAT (G) and eWAT (H) from Adipoq-TurboID^KDEL^ mice after 15 weeks of HFD feeding. Vinculin (VINC) was used as a loading control. (I and J) Quantification of protein expression assessed by western blotting in inguinal (I) and epididymal (J) white adipose tissue (iWAT and eWAT, respectively). Data represent mean ± SEM, statistical comparisons by unpaired *t* test (*, *p* < 0.05; **, *p* < 0.01). (K) Representative TMT channel assignment for a 16-plex LC-MS/MS/MS experiment compares proteomic changes induced by 15-week HFD vs. chow in iWAT or eWAT adipocytes from Adipoq-TurboID^KDEL^ Cre+ and Cre− mice (technical replicates not shown). The number of proteins passing the ROC cutoff in each experiment is also shown. (L and M) Volcano plots show log_2_(fold-changes) in secretory pathway protein expression in iWAT (L) and eWAT (M) samples following HFD feeding for 15 weeks relative to basal. Dashed lines represent cutoffs of *p* value <0.05 and fold-change >1.5. (N) Gene set enrichment analysis (GSEA) of biological processes differentially impacted by HFD and adipocyte source depot. Red color is enriched in upregulated genes, and blue is enriched in downregulated genes (*p* value <0.05, FC > 1.5).

We next investigated how DIO, a state of positive energy balance during weight gain, alters the adipocyte secretome. Adipoq-TurboID^KDEL^ mice were placed on a high-fat diet (HFD) for 6 or 15 weeks (Figure 4A) to recapitulate early-stage and advanced-stage obesity, where the 6-week timepoint reflects more moderate obesity and insulin resistance, while the 15-week timepoint represents more advanced pathology. We then characterized the ER proteome of two different adipocyte types derived from anatomically distinct white adipose tissue (WAT) depots, known to undergo hypertrophy and hyperplasia on an HFD: subcutaneous inguinal (iWAT) and visceral epididymal WAT (eWAT). We also characterized the plasma proteome in these animals at the same timepoints.

Mice fed a 60% HFD for 6 weeks (Figure S4A) or 15 weeks (Figure 4B) showed significant weight gain compared to chow-fed littermates, independent of TurboID genotype. All mice received biotin in drinking water (1.5 mg/mL) for 7 days prior to tissue and plasma collection for MS analysis, and no differences in biotin-water intake were observed compared to Cre-negative controls (Figures 4C and S4B). As expected, mice fed HFD at both timepoints exhibited higher fasted glucose (Figures 4D and S4C), impaired glucose disposal during a glucose tolerance test (GTT) (Figures 4E and S4D), and elevated fat mass (Figure 4F) compared to chow-fed controls.

We confirmed TurboID expression by V5 western blotting and verified robust proteome biotinylation in bulk iWAT and eWAT from mice at 6 and 15 weeks of DIO (Figures 4G–4J; Figures S4E and S4F). We next compared the expression levels of adipocyte proteins known to change in abundance in DIO mice by performing western blots on whole tissue extracts. Consistent with prior reports, we observed alterations in the levels of the following proteins in tissue derived from the 15-week DIO group: complement factor D (CFAD or adipsin), LRG1, fatty acid binding protein 4 (FABP4), and retinol-binding protein 4 (RBP4)^38,44–46^ (Figures 4G–4J).

We then analyzed the biotinylated proteomes from iWAT and eWAT lysates from 15-week HFD-fed mice using TurboID-TMT, enabling comparisons within each depot across two 16-plex experiments of animals on chow and HFD (Figure 4K; technical replicates not shown). Using experiment-specific ROC cutoffs, we quantified more than 2,300 unique proteins in both iWAT and eWAT samples at 15 weeks of chow vs. HFD feeding (Figure 4K; Table S1). Among these, 668 iWAT proteins and 809 eWAT proteins showed significant diet-specific regulation (*p* < 0.05, FC > 1.5; Figures 4L and 4M). GO term analysis revealed proteins reduced in the DIO samples were associated with glycosylation and ECM formation in both adipocyte types (Figure 4N). In the 15-week HFD samples, 80 proteins associated with vesicle-mediated transport were specifically upregulated in eWAT adipocytes, while in iWAT adipocytes, there was downregulation of genes associated with immune system processes not seen in eWAT samples.

More than 300 proteins were significantly elevated in each depot in DIO mice, including leptin (LEP; 2.2 log_2_FC in iWAT, 1.2 log_2_FC in eWAT), PLIN4 (1.4 log_2_FC in iWAT, 0.5 log_2_FC in eWAT), follistatin-related protein 1 (FSTL1; 2.4 log_2_FC in iWAT, 5.2 log_2_FC in eWAT), and serpin peptidase inhibitor 1 (PEDF; 1.3 log_2_FC in eWAT). We also detected decreased levels of CFAD (−4.5 log_2_FC in iWAT, −2.7 log_2_FC in eWAT) and resistin (RETN; −0.8 log_2_FC in iWAT, −3.0 log_2_FC in eWAT), a hormone involved in insulin resistance (Figures 4L and 4M). Additional factors previously annotated as obesity-regulated were also identified, including SMOC1 (−0.3 log_2_FC in iWAT, −1.9 log_2_FC in eWAT), peptidyl-glycine α-amidating monooxygenase (AMD; 1.1 log_2_FC in iWAT, 0.9 log_2_FC in eWAT), cerebral dopamine neurotrophic factor (CDNF; −0.3 log_2_FC in iWAT, −1.5 log_2_FC in eWAT), and γ-synuclein (SYUG; 1.6 log_2_FC in iWAT, 0.8 log_2_FC in eWAT). Finally, MTR1L (GPR50) was increased by 2.3 log_2_FC in iWAT and by up to 5.0 log_2_FC in eWAT, making it one of the most strongly induced proteins in both depots. MTR1L is an orphan GPCR with a sequence variant linked to elevated triglycerides and decreased HDL in obese individuals.^47^

Many of the proteins downregulated in eWAT at 15 weeks of HFD were also suppressed in mice treated with LPS, including neurotrophic factors (NENF, CDNF), metabolic (CHLE, AMPE), and inflammatory (ANGPTL2) modulators, as well as ER stress regulators (CNPY2). Overall, 107 proteins were suppressed in both conditions, representing 24.3% of all proteins suppressed after 15 weeks of HFD. These similarities between DIO and LPS treatment raise the possibility that chronic inflammation may contribute to the metabolic dysfunction associated with prolonged DIO.

### The white adipocyte tissue secretory proteome in mice fed an HFD for 6 weeks

To capture the response of adipocytes to an HFD, we characterized the adipocyte secretory proteome of iWAT and eWAT mice fed chow or HFD for 6 weeks (Figure S4G; technical replicates not shown). Western blot confirmed altered levels of CFAD, LRG1, FABP4, and RBP4 expression in both depots (Figures S4E and S4F), although consistent with prior reports, these changes were more pronounced at 15-week HFD.

Proteomic coverage was comparable across depots and diet duration, with over 2,000 proteins passing ROC cutoffs in each depot after 6 weeks on the diet (Table S1). Among these, we observed diet-specific regulation of 252 proteins in iWAT and 431 proteins in eWAT (*p* < 0.05, FC > 1.5; Figures S4H and S4I). Overall, the magnitude of proteomic changes was lower than at 15 weeks. GO analysis revealed that genes regulating protein folding were significantly increased in iWAT (Figures S4J and S4K), suggesting that feeding mice an HFD for 6 weeks is associated with ER stress in this depot. Similarly, several ER stress-related proteins were upregulated in eWAT as well (Figure S4L), although less pronounced, suggesting depot-specific differences.

Consistent with the impaired glucose tolerance at 6 weeks of HFD, we found elevated levels of obesity-associated secreted factors, including LEP (2.9 log_2_FC in iWAT, 2.4 log_2_FC in eWAT), APOA4 (1.3 log_2_FC in iWAT), and VLDLR (1.0 log_2_FC in iWAT, 1.2 log_2_FC in eWAT), and reduced levels of CFAD (−1.8 log_2_FC in iWAT, −2.5 log_2_FC in eWAT; Figures S4H and S4I). PLIN4 was upregulated in both depots in HFD-fed mice; paradoxically, this protein was also elevated in adipocytes from fasted mice, suggesting context-dependent roles of perilipin in the negative and positive energy balance states.

A subset of proteins upregulated in iWAT at 6 weeks of HFD were downregulated in eWAT at 15 weeks of DIO, as well as in the LPS-mediated negative-energy-balance model. These included the neurotrophic factor NENF, the neurotransmitter-modulating enzyme CHLE, and AMD, an enzyme that catalyzes amidation of the C-terminus of peptides, which is necessary to extend the half-life of circulating factors, increasing their affinity toward targets of interest.^48^

Lastly, to assess whether some of these protein-level changes were regulated at the transcriptional level, we performed qPCR analysis on bulk iWAT and eWAT tissues, although suboptimal due to cellular heterogeneity of bulk WAT, and found concordant diet- and depot-specific regulation of the genes encoding the following HFD-regulated proteins: *Pam* (AMD), *Ppic*, *SerpinF*, *Cdnf*, *Sncg*, *Nenf*, *Flst1,* and *Gpr50* (MTR1L) (Figure S4M).

### Depot-specific differences in DIO mice

**Figure 5.**
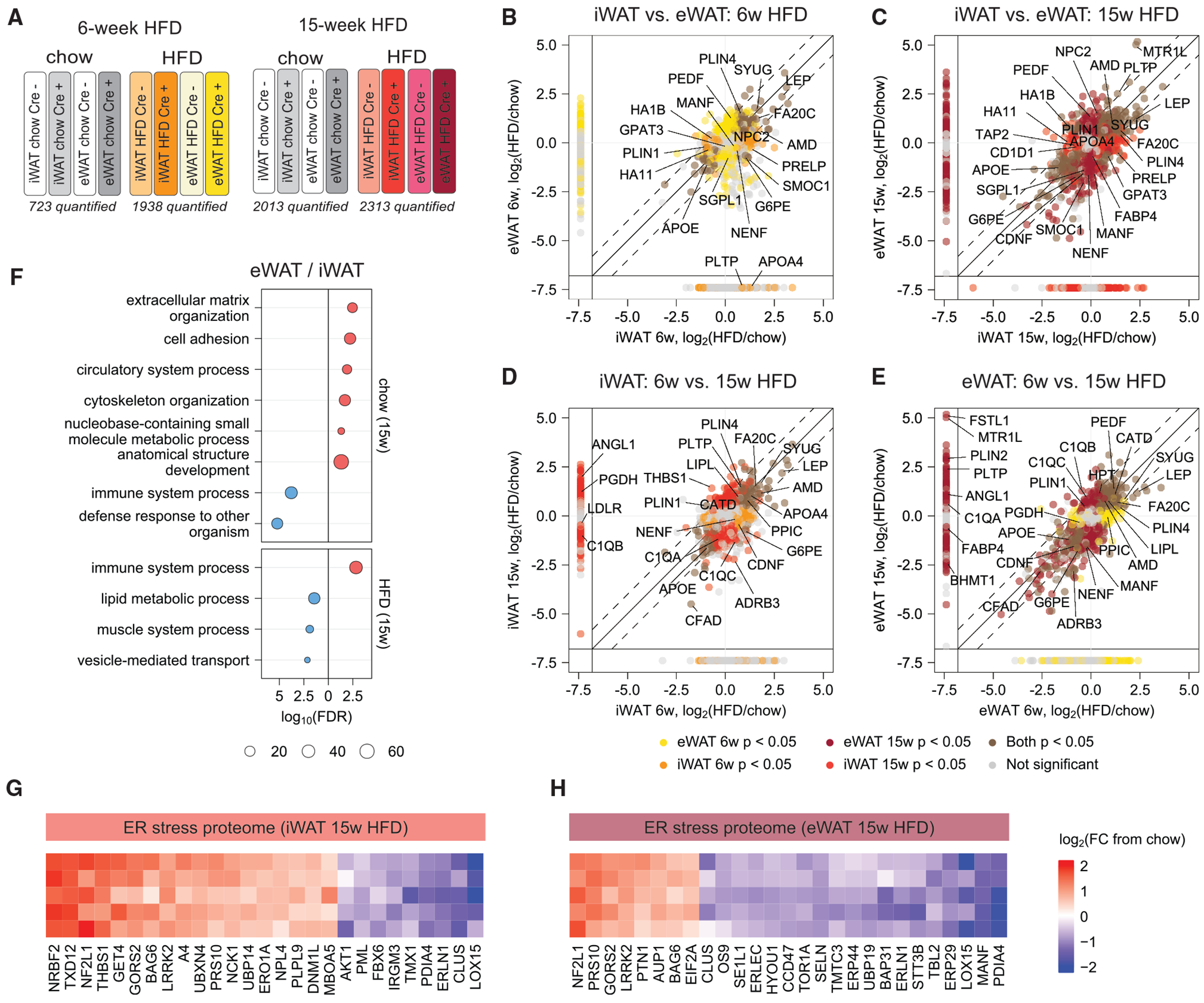
Dynamic depot-dependent reprograming of the adipocyte proteome in response to early- and late-stage DIO (A) Representative TMT channel assignments for a 16-plex LC-MS/MS/MS experiment compare iWAT and eWAT-derived adipocyte proteomes of Adipoq-TurboID^KDEL^ Cre+ and Cre− mice fed chow or HFD for 6 or 15 weeks (technical replicates not shown). The number of proteins passing the ROC cutoff in each experiment is also shown. (B and C) Correlational analysis of inter-depot differences (inguinal vs. epididymal) in log_2_(fold-changes) in protein expression at 6 weeks (B) and 15 weeks (C) of HFD feeding compared to chow. Proteins quantified in only one of the fat depots are shown on margins. (D and E) Correlational analysis of log_2_(fold-changes) in protein expression follows HFD feeding for 6 and 15 weeks in inguinal (D) and epididymal (E) adipocytes compared to chow. Proteins quantified for only one of the HFD timepoints are shown on margins. (F) Gene set enrichment analysis (GSEA) of biological processes differentially impacted by HFD in eWAT and iWAT adipocytes after 15-week HFD feeding compared to chow. Red color is enriched in upregulated genes, and blue is enriched in downregulated genes (*p* value <0.05, FC > 1.5). (G and H) Heat maps show differentially expressed proteins contributing to ER stress GO-term enrichment in iWAT (G) and eWAT (H) samples following HFD feeding for 15 weeks. Data are presented as log_2_(fold-change) compared to the chow condition.

Increased visceral adipose tissue is associated with comorbidities, while excess subcutaneous adipose tissue is comparatively benign. We investigated this by directly comparing proteomic differences between these two depots in mice fed an HFD for 6 and 15 weeks (Figures 5A–5C). Protein digests from different conditions and genotypes were split into four separate 16-plex MS experiments, which were TMT-labeled and analyzed by MS (Figures 5A and S5A; technical replicates not shown). This enabled a direct comparison of the iWAT and eWAT secretory pathway proteomes at 6 and 15 weeks under chow or HFD conditions (Figures 5B and 5C). Across experiments, we quantified between 976 and 2,373 unique proteins after ROC filters (Table S1). Combined, these proteomic datasets generated a comprehensive overview of the differences in the adipocyte secretory proteome in the response of each depot to the different diets, revealing clear depot-specific signatures (Figures 5B and 5C).

In the eWAT proteome, there was specific upregulation of the cholesterol-trafficking protein NPC2 (log_2_FC of 0.6, 0.3, 0.7, and 1.4 in 6w iWAT, 6w eWAT, 15w iWAT, and 15w eWAT, respectively) and anti-angiogenic factor PEDF (log_2_FC of 0.4, 1.2, and 1.3 in 6w iWAT, 6w eWAT, and 15w eWAT, respectively), accompanied by the downregulation of neurotrophic factor CDNF (−0.3 and −1.5 log_2_FC in 15w iWAT and eWAT, respectively), SMOC1 (−0.3 and −1.9 log_2_FC in 15w iWAT and eWAT of DIO), and sphingosine-1-phosphate lyase SGPL1 at 15 weeks of DIO (−0.3 and −1.3 log_2_FC in iWAT and eWAT). In contrast, the following proteins were specifically upregulated by DIO in iWAT, but not eWAT (Figures 5B and 5C): APOA4 (1.3 and 1.2 log_2_FC at 6 and 15 weeks, respectively), the insulin receptor-stabilizing factor CAVN2^49^ (0.9 and 0.7 log_2_FC at 6 and 15 weeks, respectively), and the multifunctional ECM protein PRELP (1.3 log_2_FC at both 6 and 15 weeks in iWAT). In the secretory proteome of iWAT, there was also an apparent coordinated suppression of antigen presentation machinery in response to DIO, not seen in the eWAT secretory proteome. This included the downregulation of the glycolipid-presenting protein CD1D1 (−0.3 and −0.6 log_2_FC at 6 and 15 weeks of DIO) and multiple MHC class I components—peptide-loading complex (TAP2, TPSN; −0.7 and −0.9 log_2_FC in 15w iWAT, respectively) and MHC class I molecules (HA11, HA1B; −1.1 and −1.1 log_2_FC in 15w iWAT, respectively).

We next compared proteomic changes between 6 and 15 weeks of DIO from separate MS experiments (Figures 5D and 5E). Many proteins were similarly altered by diet independent of duration, including CFAD, ADRB3, and LEP. However, a subset of the proteome was only altered at one timepoint and not the other with a larger number of proteins showing a greater increase at 15 weeks including the lysosomal protease CATD (0.7 and 1.0 log_2_FC in 15w iWAT and eWAT, respectively), acute-phase protein HPT (1.0 log_2_FC in 15w eWAT), and complement components (C1QA, C1QB, C1QC; 0.7, 1.3, and 0.9 log_2_FC in 15w eWAT, respectively). Many of the proteins that were upregulated at 15 weeks were not detected at 6 weeks, suggesting that they may play a role in the adaptation to increasing adiposity. These included the anti-angiogenic factor ANGL1, the pro-inflammatory cytokine FSTL1, PLIN2, and MTR1L. Proteins quantified in only a single dataset are shown on the x- and y axes (Figures 5B–5E), although their absence in the 6-week experiment prevented direct temporal comparison.

Conversely, we identified a subset of proteins specifically regulated at the 6-week timepoint or showing divergent regulation across timepoints, including an increase in G6PE at 6 weeks in iWAT, while it was significantly decreased at 15 weeks of DIO in both depots (Figures 5D and 5E). The following proteins were also elevated at 6 weeks of DIO vs. 15-week samples, including neurotrophic factors CDNF, MANF, and NENF, which were increased in iWAT adipocytes relative to chow but decreased in eWAT adipocytes (Figure S5B). Moreover, by 15 weeks of DIO, these factors were significantly decreased in eWAT adipocytes but not in iWAT, suggesting a depot-specific response to HFD. GO analysis further revealed that in eWAT adipocytes, HFD elicited a stronger increase in proteins linked with immune processes compared to iWAT, while under chow conditions, the opposite pattern was observed (Figure 5F; Figures S5C and S5D). A similar induction of proteins associated with an immune response was also observed in iWAT after LPS treatment, even though the two states are quite distinct. Finally, multiple proteins associated with ER stress were significantly altered in both depots in late-stage obesity following 15 weeks of HFD feeding (Figures 5G and 5H).

### Plasma proteins secreted by white adipocytes in early and advanced obesity

**Figure 6.**
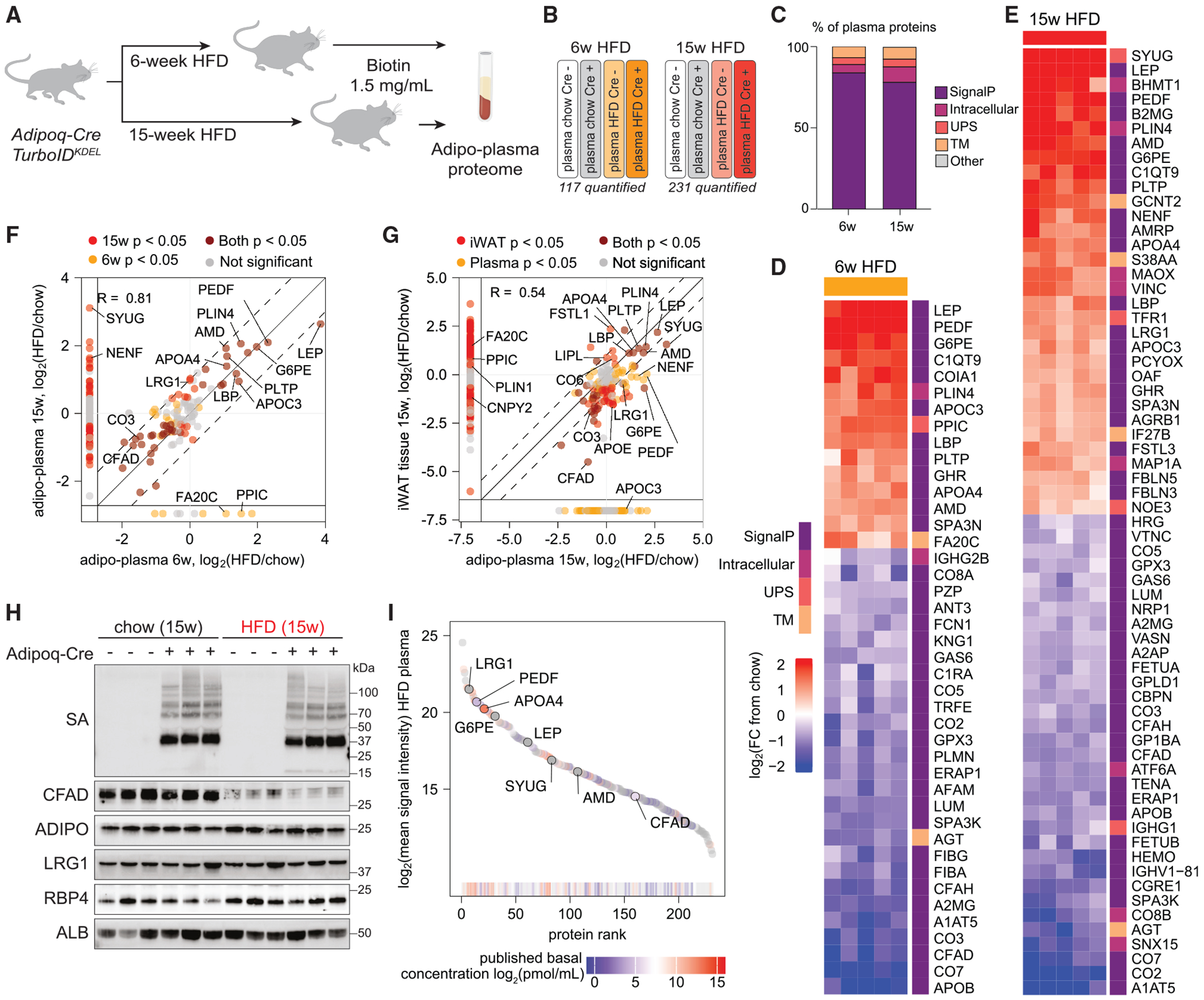
Adipo-plasma proteome in early and advanced DIO (A) Experimental design for the discovery of circulating adipokines in obese Adipoq-TurboID^KDEL^ mice after 6 and 15 weeks of HFD feeding; *n* = 5 mice per condition. (B) Representative TMT channel assignments for a 16-plex LC-MS/MS/MS experiment compare adipo-plasma proteomes of Adipoq-TurboID^KDEL^ Cre+ and Cre− mice fed chow or HFD for 6 or 15 weeks (technical replicates not shown). The number of proteins passing the enrichment cutoff in each experiment is also shown. (C) Bar plot of proteins quantified in Adipoq-TurboID^KDEL^ plasma proteome at 6 and 15 weeks of DIO, with protein class labeled based on SignalP and OutCyte predictions. (D and E) Heatmaps show Adipoq-TurboID^KDEL^ plasma proteome affected by HFD feeding in early (D) and late (E) stages of obesity. Data are presented as log_2_(fold-change) compared to the chow condition. (F) Scatterplot of log_2_(fold-changes) in adipo-plasma protein expression follows HFD feeding for 6 and 15 weeks compared to chow. Proteins quantified for only one of the HFD timepoints are shown on margins. (G) Scatterplot of log_2_(fold-changes) in protein expression in iWAT vs. plasma proteomes of Adipoq-TurboID^KDEL^ mice following HFD feeding for 15 weeks compared to chow. Proteins quantified for only one of the conditions are shown in the margins. (H) Western blot validation of protein biotinylation using streptavidin-HRP and levels of known adipokines, including CFAD, ADIPO, LRG1, and RBP4 in bulk plasma samples from Adipoq-TurboID^KDEL^ mice after 15 weeks of HFD feeding. Albumin (ALB) was used as a loading control. (I) Rank plot of mean signal intensity values of proteins quantified by TurboID-TMT in Adipoq-TurboID^KDEL^ plasma proteome following HFD feeding for 15 weeks, colored by known basal plasma concentrations.

We next performed TurboID-TMT analysis of plasma proteins secreted by white adipocytes of DIO obese mice (Figure 6A) after 6 and 15 weeks of HFD feeding (Figure 6B; technical replicates not shown). As expected, plasma samples were significantly enriched for SignalP-containing proteins, confirming selective capture of secreted proteins, including LEP, CFAD, LRG1, PLTP, as well as unconventionally secreted proteins (UPS) such as SYUG and PPIC (Figures 6C and S6A).

We identified 117 proteins from the adipo-plasma proteomes of Adipoq-TurboID^KDEL^ mice following 6 weeks and 213 proteins after 15 weeks of HFD (Table S1). Of these, 42 plasma proteins were significantly changed at 6 weeks of DIO (15 up and 27 down) and 65 were altered at 15 weeks (32 up and 33 down in HFD vs. chow) (*p* < 0.05, FC > 1.5; Figures 6D–6F). These included canonical adipocyte-derived hormones such as LEP, as well as other adipokines sensitive to nutritional state, including CFAD and its cleavage product CO3, phospholipid transfer protein PLTP, PLIN4, LBP, PEDF, and APOA4. Comparison of adipo-plasma proteomes between early- and late-stage obesity (Figure 6F) revealed that a subgroup of known adipokines was elevated in the samples from mice fed an HFD for 15 weeks, including LEP, PEDF, CFAD, and G6PE. However, a smaller number of proteins was detected at the earlier stage. These included proteins specifically detected at 6 weeks of HFD feeding, such as FA20C, and some mediators, such as SYUG and BHMT1, which were only increased at 15 weeks of DIO and were not detected at the earlier timepoint (Figure 6F; see also Table S1).

Consistent with our previous work, we confirmed LRG1 as an obesity-induced adipokine^38^ (Figure 6E). Moreover, SYUG, similar to LEP, was one of the most upregulated proteins in adipocyte-derived plasma in obese mice at 15 weeks (Figure 6E), consistent with its upregulation in both iWAT and eWAT at this timepoint (Figures 6G and S6B). We also detected increased levels of G6PE in plasma following HFD feeding, which was distinct from its decreased cellular levels in inguinal adipocytes in obesity and fasting (Figure 6G), suggesting the post-translational regulation of this circulating factor.

We validated the biotinylation of circulating proteins in plasma, as well as diet-induced regulation of known adipokines by western blot analysis of plasma proteins from 6- and 15-week chow and HFD fed mice, and, as expected, saw an impact of HFD on CFAD levels in the chronic DIO group (15 weeks; Figure 6H) compared to 6-week fed animals (Figures S6C and S6D). Globally, analysis of the dynamic range based on signal intensity and protein rank suggested we were able to cover a wide range of abundance, including hormones and soluble factors, known to circulate in much lower concentrations than more abundant plasma proteins such as apolipoproteins, CFAD, or LRG1 (Figure 6I).

Notably, our experimental design enabled direct comparison of secretory pathway changes across multiple negative and positive energy-balance models (Figures S6E–S6G). This revealed both concordant effects on proteins such as G6PE, SPA3C, and CFAD, as well as discordant regulation of proteins including GHR and B2MG across fasting, LPS, and DIO conditions.

### Secreted factors impacted by energy balance exhibit human disease associations

To investigate the potential clinical relevance of adipocyte-derived secreted proteins, we next asked which factors altered by fasting, LPS, and obesity are associated with human phenotypes in the UK Biobank (UKBB), the largest plasma proteome-phenome atlas available to date. This database includes information from 53,026 individuals with Olink-based quantification of plasma proteins across 406 prevalent diseases, 660 incident diseases, and 986 health-related traits.^50^ This resource provides statistically powerful associations between basal plasma levels of a given circulating protein and prevalence of specific clinical traits and disease outcomes in human subjects.

**Figure 7.**
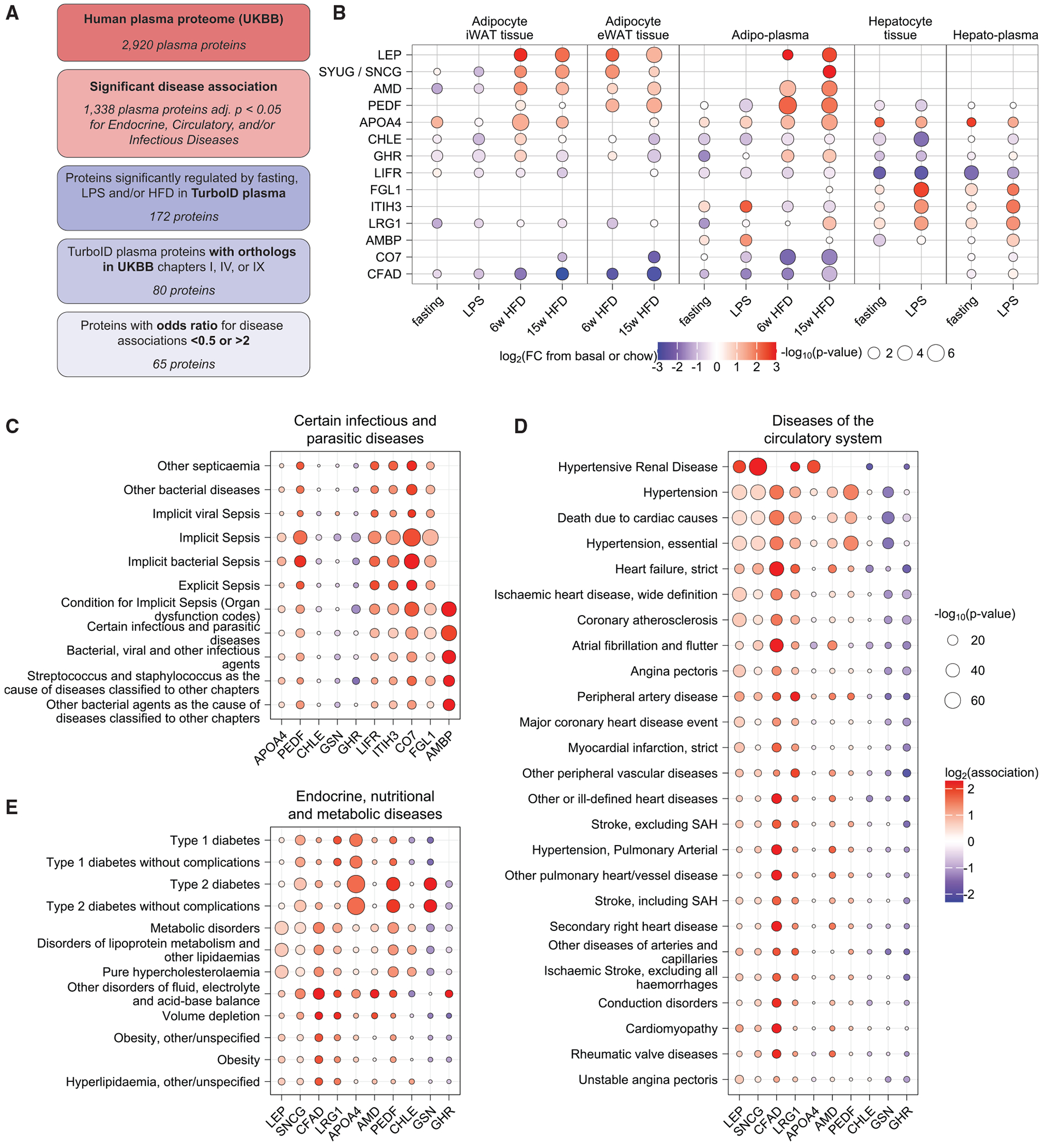
Energy balance-dependent circulating proteins are associated with human health and disease (A) Schematic shows the selection of TurboID plasma proteins with orthologs in relevant disease-associated human plasma proteins identified in a UKBB cohort of 53,026 individuals. (B) Dot plot of log_2_FC and *p* values of selected proteins detected in adipocytes, hepatocytes, and plasma across negative and positive energy balance conditions. Data are shown relative to the own control group (e.g., fasting vs. basal, LPS vs. basal, and HFD vs. chow). (C) Infectious disease associations for top-regulated hepatokines and adipokines, based on basal plasma levels of these proteins and clinical outcomes in the UKBB cohort of 53,026 individuals. (D and E) Disease associations for top-regulated adipokines, based on basal plasma levels of these proteins and clinical outcomes in the UKBB cohort of 53,026 individuals.

We identified 65 proteins that were significantly changed in the adipo- or hepato-plasma proteome under low- or high-energy balance and that were associated with endocrine, circulatory, and/or infectious diseases (Figures 7A–7E; Table S4). We also found significant associations in the UKBB cohort between obesity-regulated proteins and susceptibility to a range of infectious diseases, including LIFR, CO7, and PEDF (Figures 7B and 7C; Figures S7A–S7C). Further, a subset of circulating proteins whose levels were altered by changes in energy balance exhibited strong associations and elevated odds ratios (ORs) of circulatory and endocrine/metabolic diseases, including LEP, SYUG, LRG1, PEDF, APOA4, and CHLE (Figures 7D, 7E, and S7D–S7H). Notably, type 2 diabetes (T2D) was among the strongest associations by adjusted *p* values for SYUG (*p* = 3.66 × 10 ^−31^, OR of 1.64), PEDF (*p* = 3.47 × 10 ^−40^, OR of 4.5), and APOA4 (*p* = 1.23 × 10 ^−76^, OR of 3.23) (Figures 7E; Figures S7D, S7F, and S7G), while, as expected, LEP was associated with multiple outcomes linked with obesity such as hypertension (*p* = 1.84 × 10 ^−47^, OR of 1.4), metabolic disorder (*p* = 6.9 × 10 ^−39^, OR of 1.5), and several adverse cardiometabolic outcomes.

Among these associations, we were particularly intrigued by human γ-synuclein (SYUG), which in our experimental data is an adipocyte-enriched protein, reduced during fasting and after LPS treatment in inguinal fat cells and increased in obesity in both fat depots and in the plasma (Figure 7B). Interestingly, UK Biobank individuals with elevated circulating levels of SYUG exhibited higher odds of several metabolic disorders including T2D, hypercholesterolemia (*p* = 6.64 × 10 ^−14^, OR of 1.3), and disorders of lipoprotein metabolism (*p* = 8.93 × 10 ^−15^, OR of 1.3), as well as heart failure (*p* = 1.83 × 10 ^−22^, OR of 1.95) and other cardiovascular complications (Figure S7D). These data thus suggest that circulating γ-synuclein is significantly associated with impairments of cardiometabolic health.

## DISCUSSION

Maintaining metabolic homeostasis requires fine-tuned communication between cell types within a tissue and between distant organs. Disruption of these signals contributes to metabolic and inflammatory disorders. Yet, studying intercellular communication *in vivo* has remained challenging due to the lack of tools to precisely identify the tissue of origin of secreted proteins. Here, we present a systematic, tissue-resolved map of ER-resident and secretory pathway proteomes across major metabolic cell types, including adipocytes and hepatocytes at baseline and in response to fasting, LPS treatment, and DIO. In addition to defining cell type-specific secreted proteomes, our approach also revealed alterations in proteostatic processes in the ER, including protein folding and ER stress responses, which become disrupted in energy imbalance.^51^

This work was made possible by several key technological advances. First, we captured organ-specific circulating proteomes using a newly generated, genetically encoded PL mouse model with stable, Cre-dependent expression of an ER-targeted TurboID construct. This bypasses the limitations inherent in the viral transduction of PL constructs, including inconsistent gene delivery, off-target effects, and inflammatory responses.^52,53^ Our genetic approach facilitates robust and reproducible labeling of proteins within the ER or destined for secretion. Second, our quantitative proteomic framework, based on multiplexed TMT labeling, improves quantitative accuracy and substantially reduces missing values compared to label-free methods,^14,54^ thereby increasing sensitivity for low-abundance proteins. Finally, our bioinformatics pipeline, including ROC-based enrichment analyses^11,16^ and GO pathway mapping, ensured robust identification of bona fide secreted proteins and biologically relevant pathways.

Our data demonstrate that metabolic stress alters ER protein homeostasis in a dynamic and tissue-specific manner. In adipocytes, our findings revealed distinct secretory pathway proteome remodeling in response to metabolic and inflammatory stress during negative energy balance induced by fasting and LPS. Fasting seems to suppress anabolic lipid metabolism and ECM remodeling based on the analysis of the proteome, while enhancing lipid mobilization pathways, suggesting adaptive responses to energy deprivation. The link between systemic ECM changes and caloric restriction has been previously demonstrated in fasted human subjects and is thought to contribute to the adaptation to reduced caloric intake.^55^

Fasting also reduced ER-resident and secreted immune proteins, likely reflecting an energy-conserving adaptation under nutrient deprivation^56^ at the cost of immune function. In contrast, LPS induced impaired lipid processing and activation of inflammatory and ER stress pathways, suggesting possible contributions of acute inflammation to metabolic dysfunction. These changes are consistent with TLR4-mediated immune activation in adipocytes^57,58^ and reprogramming of the secretory machinery toward an immunometabolic phenotype to support its activation.

Hepatic responses were equally distinct; fasting suppressed complement production and promoted metabolic adaptations, including an increase in proteins crucial for gluconeogenesis, induction of PPARα target genes such as CP4AE that drive fatty acid ω oxidation,^59^ and LPIN1, a regulator of triglyceride remodeling and lipolysis.^60^ These adaptations may help supply alternative energy substrates to peripheral organs during fasting, when glucose utilization is limited. In contrast, LPS treatment amplified NF-κB/STAT3 inflammatory signaling, elevated the hepatic acute-phase response,^61–63^ and induced proteins within the SREBP2-dependent cholesterol biosynthesis axis (SREBF2, HMGCR, SQLE, FDFT1, CYP51A1). This shift supports membrane biogenesis, lipoprotein remodeling, and receptor trafficking during inflammation, consistent with prior reports.^64^ Collectively, these findings highlight two divergent adaptations: fasting activates lipid catabolism, while LPS may initiate a distinct inflammatory metabolic shift characterized by cholesterol biosynthesis and acute phase responses.

The tissue-specific secretome atlas reported here provides a comprehensive resource for functional validation and hypothesis generation. This included G6PE, which was elevated in both inguinal adipocytes in early obesity and adipo-plasma during fasting and early and late-stage obesity. This protein has a role in cell growth, redox balance, and ER stress regulation, and its deficiency correlates with fasting hypoglycemia and enhanced insulin sensitivity.^65^ G6PE has not been previously annotated as a circulating factor, its secretion by adipocytes opens new avenues for understanding its influence on systemic metabolic regulation. Since G6PE was induced by both fasting and early obesity, adipocyte-secreted G6PE may act systemically to alleviate cellular stress and favor adipose tissue expansion. G6PE was not included for detection in the ~3,000 protein Olink panel used in the UKBB cohort, the link between circulating G6PE and human disease is currently not known.

We also found that MTR1L (GPR50), an orphan receptor, showed a striking 30-fold induction in adipocytes, particularly in visceral fat cells, during DIO. While prior studies have linked this X chromosome gene to triglyceride and HDL regulation in obese humans,^47^ the precise cellular function of MTR1L remains elusive. MTR1L was also reported in a previous proteomic study describing western diet-induced changes in bulk eWAT and mature adipocytes from that depot.^66^ Independently, MTR1L knockout mice exhibit a complex metabolic phenotype, and our findings suggest a previously unappreciated role for MTR1L in adipocytes during obesity, warranting further investigation.^67^

DIO also led to a dynamic reprogramming of the adipocyte proteome. These changes included time-dependent suppression of tissue-resident or locally secreted proteins linked with ECM organization and altered levels of proteins associated with immune system processes, protein folding, and ER stress. Importantly, many of the HFD-induced changes observed here were also detected in an independent proteomic study of eWAT and iWAT as well as mature adipocytes from these two depots.^66^ We further observed a time- and fat depot-dependent increase of three proteins previously described as neurotrophic factors when obesity develops: CDNF, MANF, and NENF. CDNF is known to negatively regulate pro-inflammatory cytokine secretion and ER stress/UPR activation.^68^ We found that CDNF was induced in inguinal adipocytes, but reduced in visceral adipocytes after 15 weeks of HFD. Consistent with this depot-dependent CDNF expression pattern, we identified additional changes in proteins involved in ER stress, including negative regulators of the UPR and integrated stress response in iWAT compared to eWAT cells. MANF has previously been demonstrated to promote iWAT browning and reduce WAT inflammation and hepatic steatosis in obese mice.^69^ Lastly, NENF is a neurotrophic factor known to regulate energy balance via the hypothalamic-peripheral axis^70^ and to modulate sympathetic neuron activity. This is consistent with our data showing that its expression is altered both during inflammation-induced anorexia^71^ and obesity. Collectively, our data suggest that iWAT may modulate its own stores of these neurotrophic factors depending on nutritional intake and energy status.

Finally, our study establishes SYUG as an adipocyte-derived circulating factor whose plasma levels are acutely and chronically modulated in response to nutritional status. SYUG is elevated in chronic obesity and diminished during fasting or LPS exposure. Although historically studied in neuronal contexts, SYUG has been recently implicated in adipocyte lipolysis via interactions with ATGL and SNARE complex formation.^72,73^ Indeed, independent studies have shown SYUG is a target of peroxisome proliferator-activated receptor gamma 2 (PPARγ2),^74^ the master regulator of adipogenesis, suggesting SYUG is an actively transcribed adipocyte-enriched gene. Notably, whole-body SYUG knockout mice were protected from DIO and diabetic complications via increased energy expenditure and improved insulin signaling, as well as lower deposition of TGs in the liver. We speculate that, in addition to its demonstrated intracellular role in adipocyte lipolysis,^72,73^ secreted SYUG may modulate systemic lipid fluxes, possibly enhancing lipid uptake and storage in the liver and muscle, thereby contributing to insulin resistance in the setting of obesity. Enrichment of *SNCG* (SYUG) mRNA levels in human WAT in obesity, together with strong associations between basal circulating SYUG levels and cardiometabolic diseases in the UKBB cohort, further underscores its potential role as a significant fat-derived endocrine mediator.

In summary, this study provides a comprehensive resource for elucidating proteomic signatures across physiological states and reveals cell type-specific responses to metabolic perturbations, providing critical insights and hypotheses to test the mechanisms governing metabolic homeostasis.

### Limitations of the study

Collectively, our research introduces a powerful genetic and proteomic framework for dissecting the complexities of cell-specific secretomes *in vivo*. However, there are several limitations worth noting. First, the breadth of MS coverage is influenced by multiple factors, including TurboID expression levels, which can vary depending on Cre-driver lines, and the inherent heterogeneity of cell populations within organs. Second, detecting extremely low-abundance proteins or polypeptides derived from non-canonical open reading frames remains challenging and will require further improvements in MS instruments and analysis pipelines. Furthermore, improvements in multiplexing technologies, including the use of recently developed 32-plex TMT reagents, may further enhance experimental throughput and statistical power for future comparative proteomic analyses. Critically, while our proteomic data effectively pinpoint candidate proteins and pathways associated with various metabolic states, rigorous functional studies are indispensable to establish causal relationships between specific secretory changes and observed metabolic phenotypes. While we report broad relationships between proteins and human disease here, we have not dissected the physiological role of individual proteins regulated in specific contexts. Proteins of interest could contribute to pathology, facilitate adaptation to a stressor, or serve as biomarkers without direct functional roles. A major challenge in future studies will be developing secondary approaches to distill long protein lists to a prioritized set of high-confidence candidates for functional studies.

While mechanistic studies are required to elucidate the roles of SYUG, MTR1L, and G6PE in metabolic homeostasis, this resource will serve as a catalyst for further research. Additionally, expanding the proteomic atlases to encompass additional metabolically active tissues (e.g., pancreas, brain, and skeletal muscle) and diverse cell types will generate a more comprehensive understanding of metabolic regulation in pathophysiology. The versatile PL platform developed here can be readily adapted to investigate cell-to-cell communication in other disease contexts or physiological states. Future research will also prioritize dissecting the intricate crosstalk between different cell types and identifying sites and mechanisms of action for secreted factors characterized here.

## RESOURCE AVAILABILITY

### Lead contact

Requests for additional information, resources, or reagents should be directed to the lead contact, Ekaterina V. Vinogradova (vinograd@rockefeller.edu), who will oversee their fulfillment.

### Materials availability

Questions and requests for mouse models generated in this study should be directed to Ken H. Loh (huaijinkenleon.loh@yale.edu) and Paul Cohen (pcohen@rockefeller.edu) and will be fulfilled upon the completion of a material transfer agreement.

## STAR★METHODS

### EXPERIMENTAL MODEL AND STUDY PARTICIPANT DETAILS

#### Mice

Experiments involving mice were approved by The Rockefeller University’s Institutional Animal Care and Use Committees (Protocol numbers 21037, 19087, and 24019-H), and Yale University’s Institutional Animal Care and Use Committee (Protocol number 2022–20445).

Mice were housed 2–5 per cage in a 12-h light/12-h dark cycle with *ad libitum* access to water and regular chow except in fasting studies where chow was restricted. We used wild-type male C57BL/6J mice (Jackson Laboratory 000664), Rosa26-Cas9 knockin (*Gt(ROSA)26Sor*^tm1.1(CAG-cas9*,-EGFP)Fezh^/J, Jackson Laboratory 024858) Albumin-Cre (B6.Cg-Speer6-ps1^Tg(Alb-cre)21Mgn^/J, Jackson Laboratory 003574). CD19-Cre (B6.129P2(C)-Cd19tm^1(cre)Cgn^/J, Jackson Laboratory 006785), Adiponectin-Cre (B6.FVB-Tg(Adipoq-cre)1Evdr/J, Jackson Laboratory 028020), and LSL-TurboID-KDEL, which was generated in the transgenic core facility at Rockefeller University. All mouse lines are on a wild-type (C57BL/6J) background. Littermates of the same sex (all male) were randomly assigned to either experimental or control groups.

#### Construct generation and genotyping

LSL-V5-TurboID-KDEL-IRES-eGFP was cloned by Gibson Assembly into the CTV plasmid (Addgene #15912) at the Asc1 restriction site for recombination into the Rosa26 locus. Transgenic knock-in chimeric mice were generated by ES cell injections into mouse blastocysts, at Rockefeller’s Transgenic and Reproductive Technology Center. Mice were backcrossed eight generations on the C57BL/6J background before additional crosses. Mice were then bred to homozygosity without any obvious developmental defects. Primers for genotyping both homozygous and heterozygous mice were:

TurboID^KDEL^ Fwd 5′ CCC GAG CCT ATC CCG CTG CTG AAC 3′

TurboID^KDEL^ Rev 5′ CCC CAA TGA TCA GCT TCA CGG GTC 3′

Rosa26 Fwd 5′ AAA GTC GCT CTG AGT TGT TAT 3′

Rosa26 Rev 5′ GGA GCG GGA GAA ATG GAT ATG 3′

#### Biotin water

Biotin-containing water for basal profiling and negative energy balance experiments was prepared by dissolving solid biotin in sterile drinking water at a concentration of 0.25 mg/mL. To solubilize a higher concentration of biotin for HFD experiments (1.5 mg/mL), biotin was first dissolved in a small volume of Trizma base (3 g biotin, 20 mL, 1 M Trizma base) and then diluted in sterile drinking water to 2 L final volume with pH returned to pH 7.5 by addition of hydrochloric acid. Mice were provided *ad libitum* access to biotin-containing water during treatment.

#### LPS treatment

Heterozygous (TurboID^KDEL^/C57BL/6J) mice, with and without expression of Adiponectin- or Albumin-Cre, were used for LPS and fasting experiments. For LPS-treated animals, a frozen aliquot of LPS (Sigma Aldrich L2880, 1 mg/mL) was diluted in sterile phosphate-buffered saline (PBS) to a final concentration of 0.1 mg/mL. This solution was injected into each animal i.p. at a concentration of 1 mg/kg while saline alone was provided as a control. All mice were 8 weeks old at the time of LPS treatment.

#### Food restriction and chow consumption measurements

For fasted animals (8 weeks old), food was removed from the hopper for 48 h, while other groups of animals had *ad libitum* access to chow. Food intake was measured daily.

#### Diet-induced obesity

Homozygous (TurboID^KDEL^/TurboID^KDEL^) mice crossed with Adipoq-Cre donors^21^ were used for diet-induced obesity studies. All mice were 8 weeks old at the start of HFD/chow diet, group-housed (3–4 per cage), maintained at RT in 12-h light:dark cycle (0700–1900) and allowed *ad libitum* access to water and food (chow or 60 kcal% high-fat diet, see STAR Methods Table). For early-stage obesity, male mice of both genotypes (Adipoq-TurboID^KDEL^ Cre+ and Cre−) were weighed, randomly allocated to diet groups (minimum *n* = 4 per condition) and sacrificed after 6 weeks of HFD feeding. An independent age-matched cohort fed HFD/chow for 6 weeks was used for glucose tolerance tests and body composition scans. For late-stage obesity, male mice were fed HFD/chow for 15 weeks starting at 7 weeks of age. Biotin was administered in drinking water for 7 days prior to plasma and tissue harvests. Biotin intake was recorded daily, and body weights were recorded weekly.

#### Metabolic phenotyping in diet-induced obese mice

For fasting glucose measurements and glucose tolerance tests (GTT) mice were housed individually and fasted for 6 h in a noise-free procedure room. Circulating levels of glucose were determined from tail-derived blood using a standard glucometer. Following fasting glucose at time 0, mice were injected i.p. with bolus glucose at 1.5 g/kg in 30-s intervals and glucose measurements were taken at 15, 30, 60, 90, and 120 min post-injection. Two measurements with two separate glucometers were taken per mouse and per time-point to ensure measurement accuracy (means are reported). Body composition (fat mass, lean mass) was assessed using rodent echoMRI.

#### Blood glucose measurements

A small incision was made on the mouse’s tail to allow collection of blood, and blood glucose levels were measured using a Contour NextEZ glucometer. Body weights and blood glucose shown in Figures 1C and 1D were measured between zeitgeber time (ZT) ZT5-6.

#### Sample collection

Plasma sample collection: Mice were anesthetized with an overdose of isoflurane and blood was collected via cardiac puncture, using EDTA coated microcentrifuge tubes (Sarstedt), and placed on ice. Tubes were centrifuged at 1,000 *g* for 20–30 min at 4°C, followed by collection of the plasma fraction and storage at −80°C until further analysis. Samples were collected between ZT5-6.

Tissue sample collection: Following blood collection, mice were perfused with PBS to minimize any non-specific residual biotinylation signal, organs were then dissected, rinsed with ice-cold PBS briefly, snap frozen in liquid nitrogen, and stored at −80°C before use. Samples were collected between ZT5-6.

Fixed tissues for validation in sections: Mice were anesthetized with isoflurane and an intracardiac perfusion and fixation was performed with 1x PBS followed by cold 4% PFA. All harvested samples were post-fixed in 4% PFA at 4°C for 24 h. Fixed tissue samples were washed with PBS for three times before subsequent steps.

### METHOD DETAILS

#### Plasma processing

For mass spectrometry, 200–400 μL of plasma was diluted to 15 mL using 1x PBS and centrifuged at 2,755 *g* for 1 h at 4°C using a 3 kDa filter (Millipore, UFC900324) twice to remove biotin, until the solution was concentrated to 0.5 mL. The final 0.5 mL solution was transferred to a new 1.5 mL tube and 0.8 mL of RIPA buffer containing protease inhibitors was added before further analysis or subsequent enrichment steps.

#### Tissue lysis for liver and spleen

Half of a single lobe of liver (~400 mg) and the entire spleen from each animal were used for analysis. Tissues were minced using scissors and one 3 mm Tungsten carbide metal bead was added to the sample in a 2.0 mL microcentrifuge tube, containing 1 mL of cold RIPA buffer (10 mM Tris, 150 mM NaCl, 1% Triton X-100, 1% sodium deoxycholate, 0.1% SDS, pH 7.5) supplemented with cOmplete protease inhibitor cocktail (Roche, 04693116001) and 1 mM PMSF. Tissues were then lysed using a Qiagen TissueLyser II (20 frequency/sec for 20 min). Samples were incubated in RIPA at 4°C with end-over-end rotation for 20-30 min to boost protein extraction yield, followed by centrifugation at 18,000 *g* for 10 min, and collection of the supernatant. Protein concentration was measured using standard DC or BCA assay and subjected to further analysis or enrichment steps described below.

#### Protein extraction from adipose tissues

Adipose tissues (iWAT, eWAT) were harvested from anesthetized, PBS-perfused mice and snap frozen in liquid nitrogen, then pulverized to a fine powder (Cellcrusher). Soluble proteins were extracted using NP-40-based RIPA buffer (1% NP-40, 150 mM NaCl, 0.5% deoxycholate, 0.1% SDS, 50 mM Tris-HCl pH 7.5) containing cOmplete protease inhibitor cocktail (Roche, 04693116001) and phosphatase inhibitor (Roche, 4906845001) tablets (1 tablet per 10 mL). Lysates were incubated with end-over-end rotation for 30 min at 4°C, followed by centrifugation at 6,200 *g* (8,000 rpm) for 20 min at 4°C, and lipid-free soluble infranatant fractions were extracted for downstream analyses.

#### Immunoblotting

##### Protocol 1

Protein concentrations were determined using Pierce BCA assay and normalized to 3 μg/μL (iWAT, eWAT) or 9 μg/μL (plasma) in XT sample buffer, boiled at 90°C for 5 min and resolved at 30 μg/well (tissue extracts) or 90 μg/well (plasma) in 12-well 4–12% Criterion XT Bis-Tris gels in MES electrophoresis buffer (Figures 1, 4, 5, 6, and 7; Figures S1, S4, and S6). Proteins were transferred onto 0.45 μm (tissues) or 0.22 μm (plasma) ImmobilonP PVDF membranes in Tris-Glycine-MeOH buffer at 30V (4°C, overnight), blocked in biotin-free SuperBlock for 1 h at RT, and incubated with Streptavidin-HRP (SA), or primary antibodies against V5 tag, CFAD, LRG1, RBP4, FABP4, ADIPOQ, ALB, and VINC, as detailed in the key resources table. HRP-conjugated secondary antibodies were used for detection with enhanced chemiluminescence in a ChemiDoc Imager. Tissue panel blots for V5 were run using multiple organs at the same concentration per well (30 μg total) to allow for side-by-side comparison. Vinculin (tissues), albumin (plasma) or Coomassie Blue were used as loading controls.

##### Protocol 2

Protein samples were combined with SDS protein loading buffer, boiled for 5 min, and resolved by SDS-PAGE (Figures 2 and 3; Figure S3). Proteins were transferred onto a nitrocellulose membranes (0.2 μm, Bio-Rad, 1620112), which were then blocked with Odyssey blocking buffer (LICOR) or 3% w/v BSA in TBST (0.1% Tween 20 in Tris-buffered saline) for 30 min. Membranes were incubated overnight at 4°C with primary antibodies diluted in 3% (w/v) BSA in TBST, followed by four 5-min washes in TBST. Following primary antibody incubation, membranes were probed with IRDye 800CW or IRDye 680RD (LICOR Biosciences) (1:10000 dilution) or HRP-conjugated secondary antibodies at RT for 1 h, then imaged using Bio-Rad ChemiDoc. Blots were quantified using Image Lab software version 6.1 (Bio-Rad). HRP-labelled blots were developed using a chemiluminescence substrate (Clarity Western ECL Substrate, Bio-Rad, 1705061). Alternatively, samples were imaged using the same channels on a Bio-Rad ChemiDoc.

For Western blot validation (Figures 3G, S2I, and S2J), proteins were resolved on 4–20% Mini-PROTEAN TGX stain-free gel (Bio-Rad, 4568095). Western blot band intensity was normalized to the total intensity of the corresponding lane in a stain-free gel image.

#### Immunofluorescence

Adipose, liver or spleen tissue was collected from anesthetized mice perfused with PBS and 4% paraformaldehyde. Liver and spleen were sunk with 30% sucrose in PBS overnight. Adipose tissue was delipidated as previously described.^83^ Samples were embedded in OCT and cut into 30 μm sections at −20°C. Sample sections were blocked in 3% BSA, 1% donkey serum containing PBST (0.1% Triton X-100, 0.05% Tween 20), followed by incubation in primary antibody dilutions for 2 h at RT. After incubation with primary antibodies, samples were washed in PBST four times and then incubated in secondary antibody dilutions for 4 h at RT in blocking buffer. Samples were then washed in PBST four times and mounted with DAPI Fluoromount (Southern Biotech).

#### Confocal microscopy

Representative sample regions of iWAT were imaged using an inverted Zeiss LSM 980 laser scanning confocal microscope with a 40× objective lens. Liver and spleen were imaged using an inverted Leica TCS SP8 X laser scanning confocal microscope with a 40× objective lens.

#### Biotinylated protein enrichment

To enrich biotinylated proteins, 100 μL of streptavidin (SA) magnetic beads (Pierce, 88816) per sample was washed twice with RIPA buffer (150 mM NaCl, 1% NP-40, 0.5% sodium deoxycholate, 0.1% SDS, 50 mM Tris pH 7.5) with protease and phosphatase inhibitors. The washed slurry was then incubated with cell lysates containing the same amount of protein input (3–5 mg of protein) for all samples of that same tissue type and incubated with end-over-end rotation at 4°C overnight. Using a magnetic rack, the beads were subsequently washed twice with 1 mL of RIPA lysis buffer, once with 1 mL of 1 M KCl, once with 1 mL of 0.1 M Na_2_CO_3_, once with 1 mL of 2 M urea (freshly prepared) in 10 mM Tris-HCl (pH 8.0), and twice with 1 mL RIPA lysis buffer. A small volume of protein-bound beads (5% of total volume) was removed at this point for validation by Western blot and bead-bound proteins were eluted by boiling at 95°C in 2X SDS loading buffer containing 20 mM DTT and 2 mM biotin for 15 min.

#### Elution of peptides from beads

To prepare proteomic samples for mass spectrometry analysis, biotinylated proteins bound to SA beads were washed with 1 mL of 50 mM Tris-HCl (pH 7.5), followed by two washes with 1 mL 2 M urea in 50 mM Tris (pH 7.5) buffer. The buffer was removed, the beads were re-suspended in 80 μL of 2 M urea in 50 mM Tris-HCl containing 1 mM DTT, 0.5 mM CaCl_2_, and 0.8 μg trypsin (Promega, V5111), followed by incubation at 25°C for 1.5 h with shaking at 1,000 rpm. After 1.5 h, samples were placed on a magnet, and the supernatant was transferred to fresh low-binding Eppendorf tubes. Streptavidin beads were then washed twice with 60 μL of 2 M urea in 50 mM Tris (pH 7.5), and the washes were combined with the initial supernatant for subsequent reduction, alkylation, and digestion steps.

#### Reduction, alkylation, trypsin digestion, and desalting of peptides

Samples (200 μL total) were reduced with DTT (final concentration: 8 mM; 65°C, 20 min) and alkylated with iodoacetamide (final concentration: 20 mM; 37°C, 30 min in the dark with shaking at 1,000 rpm). An additional 0.5 μg of trypsin was added to each sample and protein digestion was carried out overnight at 25°C with shaking at 700 rpm. After overnight digestion, the sample was acidified to pH 3 by adding formic acid (FA) to a final 1% (vol/vol) FA. Samples were desalted using C18 StageTips (Pierce, 87784). C18 StageTips were first conditioned with 100 μL of 100% methanol, followed by 100 μL of solvent 1 (50:50 acetonitrile (ACN): H_2_O, 0.1% FA), and twice with 200 μL of buffer A (95% H_2_O, 5% ACN, 0.1% FA). Acidified peptides were loaded onto the conditioned StageTips and washed twice with 200 μL of buffer A (95% H_2_O, 5% ACN, 0.1% FA). Samples were eluted from the StageTips with 2 × 200 μL of buffer B (80% ACN/H_2_O, 0.1% FA), and the solvent was removed until completely dry using SpeedVac vacuum concentrator.

#### TMT labeling for multiplexed quantitative proteomics

Peptides were resuspended in 100 μL of 30% ACN in 200 mM EPPS (pH 8.0). Samples were vortexed, spun down, and sonicated in a water bath for 5 min. After brief centrifugation, 3 μL of the corresponding TMTpro 16-plex reagent (Thermo Fisher, A44521) was added to each tube (20 μg/μL in dry ACN). The samples were vortexed, spun down, and left at room temperature for 1 h. To quench the labeling, 3 μL of 5% hydroxylamine was added to each sample. Samples were vortexed, spun down, and left at room temperature for 15 min. Samples were then acidified with 5 μL of FA, combined into two low binding 1.5 mL Eppendorf tubes, and dried using SpeedVac vacuum concentrator, leaving gel-like salt residues.

#### Desalting of pooled multiplexed samples

Following TMT labeling, samples were resuspended in 500 μL of buffer A (95% H_2_O, 5% ACN, 0.1% FA). An additional 20 μL of 20% FA was added to ensure the sample remained acidic. The Sep-Pak C18 cartridge was conditioned by adding 1 mL 100% ACN three times and equilibrated using 1 mL buffer A (95% H_2_O, 5% ACN, 0.1% FA) three times. Samples were loaded on the column slowly at a rate of 1 drop/sec, and the flow through was collected and re-loaded a second time. To desalt the samples, 1 mL of buffer A (95% H_2_O, 5% ACN, 0.1% FA) was passed through the cartridge three times. Samples were eluted by adding 1 mL of buffer B (80% ACN, 20% H_2_O, 0.1% FA). The eluate was then dried using SpeedVac vacuum concentrator.

#### Pre-fractionation by HPLC

Dried desalted samples were resuspended in 500 μL buffer A (95% H_2_O, 5% ACN, 0.1% FA) and loaded on a high-pressure liquid chromatography (HPLC) for offline pre-fractionation. Samples were separated on a C18 column by reverse phase HPLC, starting in a high pH buffer containing 10 mM ammonium bicarbonate over an increasing acetonitrile gradient (0–100%). Briefly, the peptides were eluted onto a Zorbax column (Extend-C18, 3.5 mm, 4.6 × 250 mm) at 0.5 mL/min flow rate and separated using the following gradient: 100% buffer C from 0 to 2 min, 0%–13% ACN from 2 to 3 min, 13%–50% ACN from 3 to 60 min, 50%–80% ACN from 60 to 70 min, 100% ACN from 71 to 75 min, 100%–0% ACN from 75 to 76 min, 100% buffer C from 76 to 85 min, 0%–13% ACN from 85 to 88 min, 13%–80% ACN from 88 to 90 min, 80% ACN from 90 to 95 min, 100% buffer C from 96 to 101 min, 0%–13% ACN from 101 to 104 min, 13%–80% ACN from 104 to 106 min, 80% ACN from 106 to 111 min, and 80%–0% ACN from 111 to 112 min (buffer C: 10 mM aq. NH_4_HCO_3_). The receiving plate contained 10 μL 20% FA in each well to acidify eluting peptides and the collection was set to a time-dependent mode. Samples were collected into a deep 96-well plate, pooled across elution times (12 fractions for tissue, 4 fractions for plasma), and then dried using SpeedVac vacuum concentrator. Because the adipo-plasma and hepato-plasma proteomes were expected to be lower in abundance than the intracellular ER-associated proteomes, we pooled HPLC-fractionated samples into four final fractions to optimize coverage and enhance detection of low abundance factors.

#### Liquid chromatography-tandem mass spectrometry analysis (LC-MS/MS/MS)

Samples were re-suspended in 10 μL of buffer A and briefly sonicated before analysis by liquid chromatography-tandem mass spectrometry (7.5 μL injection) using Orbitrap Eclipse mass spectrometer (Thermo Fisher) with UltiMate 3000 Series Rapid Separation LC system. Peptides were eluted onto a 2 μm, 100 Å , 75 μm × 25 cm capillary column at 0.25 μL/min flow rate and separated using the following gradient: 5% LC-MS buffer B (ACN, 0.1% FA) in LC-MS buffer A (H_2_O, 0.1% FA) from 0 to 10 min, 5%–20% buffer B from 20 to 120 min, 20%–45% buffer B from 120 to 140 min, 45%–95% buffer B from 140 to 145 min, 95% buffer B from 145 to 147 min, 5% buffer B from 147 to 149 min, 95% buffer B from 149 to 151 min, 5% buffer B from 151 to 160 min).

The following scan sequence was used: (1) MS1 master scan (Orbitrap analysis, resolution 120,000, scan range 400-1,600 m/z, RF lens 40%, normalized AGC Target 250%, automatic maximum injection time, profile mode with enabled dynamic exclusion (repeat count 1, duration 60 s); (2) Selection of top 20 ions for MS2 analysis; (3) MS2 analysis, which consisted of quadrupole isolation (isolation window 0.7 m/z) of precursor ion followed by collision-induced dissociation (CID) in the ion trap (fixed collision energy mode, normalized collision energy 35%, maximum CID activation time 10 ms, Activation Q 0.25). Except for diet-induced obese and chow-fed iWAT and eWAT analyses, all runs used real-time search with a 2017 mouse FASTA database and included static modifications for cysteine carbamidomethylation (Δ Mass 57.0215), lysine-, and *N*-terminal amine modification with TMTpro 16-plex reagents (Δ Mass 304.2071), as well as variable modifications for methionine oxidation (Δ Mass 15.9949). Maximum missed cleavages were set to 2, with maximum variable modifications per peptide at 1.

#### RNA preparation and quantitative PCR

Total RNA was extracted from white fat tissue, spleen or liver using TRIzol (Invitrogen) along with QIAGEN RNeasy mini kits. For quantitative PCR (qPCR) analysis, RNA was reverse transcribed using the QuantiTect Reverse Transcription Kit (Qiagen). cDNA was used in qPCR reactions containing SYBR-green, fluorescent dye (ABI). Relative mRNA expression was determined by normalizing to the geometric mean of *Ppib* (cyclophilin B) and *Tbp* (TATA-box binding protein) levels using the ΔΔC_t_ method. Primer sequences are:

*Cyclo*/*Ppib* Fwd 5′ GGA GAT GGC ACA GGA GGA A 3’

*Cyclo*/*Ppib* Rev 5′ GCC CGT AGT GCT TCA GCT T 3’

*Tbp* Fwd 5′ GGG TAT CTG CTG GCG GTT T 3’

*Tbp* Rev 5′ TGA AAT AGT GAT GCT GGG CAC T 3’

*Pam* Fwd 5′ CTG GGG TCA CAC CTA AAG AGT 3’

*Pam* Rev 5′ ATG AGG GCA TGT TGC ATC CAA 3’

*Ppic* Fwd 5′ TGA CGG ACA AGG TCT TCT TTG A 3’

*Ppic* Rev 5′ CAG AGC CAC GAA GTT TTC CAC 3’

*Serpinf1* Fwd 5′ GCC CTG GTG CTA CTC CTC T 3’

*Serpinf1* Rev 5′ CGG ATC TCA GGC GGT ACA G 3’

*Cdnf* Fwd 5′ CTT TTG CGC CGG GTT TTG TAT 3’

*Cdnf* Rev 5′ AGG GAG TTG TAG AAT CGG TCT AA 3’

*Sncg* Fwd 5′ AAA GAC CAA GCA GGG AGT AAC G 3’

*Sncg* Rev 5′ GAC CAC GAT GTT TTC AGC CTC 3’

*Nenf* Fwd 5′ GAA GGG AGT GGT GTT CGA TGT 3’

*Nenf* Rev 5′ GTG TCG TGA GTG AGG TCT GC 3’

*Flst1* Fwd 5′ CAC GGC GAG GAG GAA CCT A 3’

*Flst1* Rev 5′ TCT TGC CAT TAC TGC CAC ACA 3’

*Gpr50* Fwd 5′ AGA GCA ACA TGG GAC CTA CAA 3’

*Gpr50* Rev 5′ GCC AGA ATT TCG GAG CTT CTT G 3’

### QUANTIFICATION AND STATISTICAL ANALYSIS

#### Statistical analyses

Statistical analyses were performed using GraphPad Prism and R packages. The results of statistical analyses for each experiment can be found in corresponding figure legends. All values are reported as mean ± SEM. P-values below 0.05 were considered significant throughout the study.

#### Data processing for proteomic experiments

Raw data files were processed using RAW converter (available at github.com/proteomicsyates/RawConverter) and the resulting MS1, MS2, and MS3 files were uploaded to the Integrated Proteomics Pipeline (IP2). Peptide identification was performed against a reverse concatenated, non-redundant version of the Mouse Uniprot database (release-2017_07) using the ProLuCID algorithm. Static modifications for TMT labeling were set for *N*-termini and lysine residues (+304.2071 Da for TMT 16-plex). Search results were filtered with DTASelect (v2.0) to achieve a peptide-level false discovery rate below 1%. MS3 reporter ions were used for quantification with a mass tolerance of 20 ppm via the IP2 platform.

#### Filtering and normalization of proteomic data

Experiments were processed individually with the following filters: removal of non-unique peptides, removal of half-tryptic peptides, removal of peptides with more than two internal missed cleavages, removal of peptides with low (<5,000) average of reporter ion intensities for all pairs of channels, and peptides with high variation between all pairs of channels (coefficient of variation >0.5). Additionally, keratin proteins and proteins whose human orthologs were quantified in over 200 experiments in the CRAPome^43^ database were labeled as contaminants and excluded from analysis.

Median normalization was performed on signal intensities for Cre+ channels within each experiment. True positive proteins (defined in Receiver operating characteristic analysis) with 2+ peptides were used to calculate the median per channel and normalization factors were calculated by dividing the median of all Cre+ channel medians by each individual channel’s median. Each Cre+ channel was then multiplied by its corresponding normalization factor.

#### Receiver operating characteristic (ROC) analysis

For tissue samples, receiver operating characteristic (ROC) curves were used to set the TMT ratio cutoffs that would maximize the retention of true positives, while minimizing the retention of false positives in each case, as described by the Ting Lab.^11,16^

Proteins were labeled as true positive based on subcellular location annotations retrieved from uniprot.org. Proteins with any of the following annotations were labeled as true positive (case-insensitive): “secreted”, “endoplasmic reticulum”, “rough endoplasmic reticulum”. Proteins were labeled as false positive if they were found in the human mitomatrix list from Table S1 from Rhee et al., 2013.^84^ Additionally, proteins with signal peptide annotation were removed from the false positive list. Proteins labeled as both true positive and false positive through this approach were reassigned as true positive.

#### Gene Ontology term enrichment analysis

Gene ontology enrichment analysis was performed on the significant upregulated or downregulated proteins (fold-change >1.5 and *p*-value <0.05) using Fisher’s exact test implemented in GOATOOLS against the Gene Ontology (goslim_generic.obo, downloaded on April 24, 2024). An experiment-specific background (i.e., all proteins quantified in the experiment) was used for all analyses except for the cross-experiment analysis (Figure S2F) where the full mouse proteome was used. GO Biological Process terms were mapped to GOslim terms using the GOATOOLS mapslim.py script. After analysis, terms with a Benjamini-Hochberg corrected *p*-value less than 0.05 were retained.

#### Absolute plasma concentrations

Concentration of plasma proteins quantified by multiple reaction monitoring mass spectrometry in Michaud et al. (2018)^42^ were compared with proteins detected in the present study. Of the 385 target analytes detected in C56BL/6J or C57BL/6BR mice in supplementary tables 4, 6, and 7 of Michaud et al. (2018), 243 were detected in one or more of the basal, negative energy balance, and obesity conditions. Values were displayed for comparison in Figures 3, 6, S3 and S6, and are available in Tables S2 and S3.

#### UK Biobank

UK Biobank protein-disease associations for prevalent diseases were retrieved from the protein phenome atlas^50^ (proteome-phenome-atlas.com). P-values for diseases were corrected through the Bonferroni correction and only significant associations (Bonferroni adjusted *p* < 0.05) are shown. Proteins with significant disease associations in UKBB chapters I, IV or IX orthologous to TurboID plasma proteins significantly regulated by low- or high-energy balance are listed in Table S4.

## Supplementary Material

1

2

3

4

5

Supplemental information can be found online at https://doi.org/10.1016/j.celrep.2026.117507.

## Figures and Tables

**Table:** KEY RESOURCES TABLE

REAGENT or RESOURCE	SOURCE	IDENTIFIER/RRID	
Antibodies			
Streptavidin, HRP-linked	Cell Signaling	Cat. 3999S	AB_10830897
anti-V5 tag	Abcam	Cat. ab206562	AB_3719924
anti-Human IgG, HRP-linked	Abcam	Cat. ab6858	AB_955433
anti-mAcrp30 (adiponectin)	R&D Systems	Cat. AF1119	AB_2221770
anti-Goat IgG, HRP-linked	Thermo Fisher	Cat. A15999	AB_2534673
Streptavidin, Alexa 647	BioLegend	Cat. 405237
anti-mAlbumin	Cell Signaling	Cat. 4929S	AB_2225785
anti-Rabbit IgG, HRP-linked	Cell Signaling	Cat. 7074S	AB_2099233
anti-BTK	Cell Signaling	Cat. 8547S	AB_10950506
anti-Ces1d	Santa Cruz Biotechnology	Cat. sc-374160	AB_10988772
IRDye 800CW Goat anti-Mouse IgG Secondary	LI-COR	Cat. 926-32210	AB_621842
anti-Complement C6	Invitrogen	Cat. PA5-117369	AB_2901999
IRDye 680CW Goat anti-Rabbit IgG Secondary	LI-COR	Cat. 926-68071	AB_10956166
anti-SerpinA3N	R&D Systems	Cat. AF4709	AB_2270116
IRDye 680RD Donkey anti-Goat IgG Secondary	LI-COR	Cat. 926-68074	AB_10956736
IRDye 800CW Streptavidin	LI-COR	Cat. 926-32230
anti-mFactor D (Adipsin, CFAD)	R&D Systems	Cat. AF5430	AB_1655868
anti-LRG1	Sigma-Aldrich	Cat. HPA001888	AB_1079276
anti-FABP4	R&D Systems	Cat. AF1443-SP	AB_2102444
anti-mRetinol binding protein 4	R&D Systems	Cat. AF3476	AB_2167682
anti-Sheep IgG, HRP-linked	Thermo Fisher	Cat. A16041	AB_2534715
anti-Vinculin	Cell Signaling	Cat. 4650S	AB_10559207
Chemicals, peptides, and recombinant proteins		
Biotin	Sigma-Aldrich	Cat. B4501
NP-40	Millipore	Cat. 492016
cOmplete Protease Inhibitor Cocktail tablets	Roche	Cat. 04693159001
PhosphoSTOP	Roche	Cat. 04906837001
XT MES Buffer	Bio-Rad	Cat. 1610789
Criterion 4–12% Bis-Tris 12-well gels	Bio-Rad	Cat. 3450123
Tris/Glycine transfer Buffer	Bio-Rad	Cat. 1610771
SuperBlock T20 blocking buffer	Thermo Scientific	Cat. 37536
0.45 μm PVDF membrane	Millipore	Cat. IPVH00010
Enhanced Chemiluminescence (ECL)	PerkinElmer	Cat. NEL104001EA
Paraformaldehyde	Sigma-Aldrich	Cat. P6148
Dichloromethane	Fisher Scientific	Cat. AA39116K2
Hydrogen peroxide, 30%	Fisher Scientific	Cat. H325100
Heparin	Sigma-Aldrich	Cat. H3393
Dibenzyl ether	Sigma-Aldrich	Cat. 108014
Pierce Streptavidin Magnetic Beads	Thermo Scientific	Cat. 88817
Urea	Supelco	Cat. 108487
Iodoacetamide	Sigma-Aldrich	Cat. I1149
Dithiothreitol (DTT)	Fisher Scientific	Cat. BP172-25
Trypsin, sequencing grade	Promega	Cat. V5111
Acetonitrile, LC/MS grade	Fisher Scientific	Cat. A955
Hydroxylamine solution	Sigma-Aldrich	Cat. 467804
EPPS	Alfa Aesar	Cat. A13714-22
TMTpro 16-plex Label Reagent Set	Thermo Scientific	Cat. A44521
Lipopolysaccharide (LPS)	Sigma-Aldrich	Cat. L2880
Nitrocellulose membrane, 0.2 μm	Bio-Rad	Cat. 1620112
Intercept (TBS) Blocking Buffer	LI-COR	Cat. 927-60001
D−(+)-Glucose	Sigma-Aldrich	Cat. G8270
Critical commercial assays		
DC assay kit	Bio-Rad	Cat. 5000111
BCA assay kit	Thermo Scientific	Cat. 23228
Pierce C18 pipette tips	Thermo Scientific	Cat. 87784
Sep-Pak Vac C18 cartridges	Waters	Cat. WAT054955
Glucose Meter, Glucose Strips	Nova Max Plus Blood Glucose Monitoring System	Cat. 8548043524
QuantiTect Reverse Transcription Kit	QIAGEN	Cat. 205311
SYBR GreenER qPCR SuperMix	Invitrogen	Cat. 11760500
Deposited data		
All raw proteomic data has been uploaded to PRIDE	This paper	PRIDE: PXD075539
Experimental Models: Organisms/strains		
TurboID^KDEL^	This paper	
Albumin-Cre (B6.Cg-Speer6-ps1^Tg(Alb-cre)21Mgn^/J)	The Jackson Laboratory	Strain 003574
Adiponectin-Cre (B6.FVB-Tg(Adipoq-cre) 1Evdr/J)	The Jackson Laboratory	Strain 028020
CD19-Cre (B6.129P2(C)-Cd19^*tm1(cre)Cgn*^/J)	The Jackson Laboratory	Strain 006785
Wild-type (C57BL/6J)	The Jackson Laboratory	Strain 000664
Recombinant DNA		
CTV plasmid	Addgene	Cat. 15912
Software and algorithms		
TurboID analysis pipeline	This paper	github.com/vinogradova-lab/cell-type-specific-proximity-labeling
IP2 and ProLuCID	Integrated Proteomics Applications	Xu et al., *J Proteomics* (2015)^75^
RawConverter (v1.2.0.1)	Yates Lab, Scripps Research	He et al., *Anal Chem* (2015)^76^
NumPy (v1.22.4)	Van der Walt et al., 2010^77^	numpy.org
Pandas (v1.4.3)	McKinney, 2010^78^	pandas.pydata.org
ggplot2 (v3.3.5)	Wikham, 2016^79^	ggplot2.tidyverse.org
SignalP (v6.0)	Emanuelsson, 2007^25^	services.healthtech.dtu.dk/services/SignalP-6.0/
ComplexHeatmap (v2.10.0)	Gu, 2022^80^	github.com/jokergoo/ComplexHeatmap
GOATOOLS (v1.3.1)	Klopfenstein et al. 2018^81^	github.com/tanghaibao/goatools
scikit-learn (v1.0.2)	Pedregosa et al., 2011^82^	scikit-learn.org/stable
ggrepel (v0.9.1)	github.com/slowkow/ggrepel	
Image Lab Software (v6.1)	Bio-Rad	
Other		
Rodent regular chow diet	PicoLab	Cat. 5053
Rodent diet with 60 kcal% Fat	Research Diets	Cat. D12492
Rodent water bottle nozzle, 30mm, short straight	Vision Racks & Cages	Type E
Microvette EDTA capillary blood collection tubes	Sarstedt	Cat. 16.444.100

## Data Availability

Raw proteomic data and the code used for data processing and visualization have been deposited to PRIDE (PXD075539) and GitHub (github.com/vinogradova-lab/cell-type-specific-proximity-labeling) and are publicly available as of the date of publication. Any additional information required to reanalyze the data reported in this paper is available from the lead contact upon request.

## Highlights

- Genetically encoded TurboID^KDEL^ enables cell-type-specific secretome labeling
- Metabolic stress alters ER proteostasis in a dynamic and cell-specific manner
- ER stress and ECM proteomes are differentially affected by energy state
- Cell-type-specific secretome labeling identifies plasma markers of human disease
