# Supplementary material for: Cell-type-specific proximity labeling of organ secretomes reveals energy balance-dependent proteomic remodeling
Source: Cell Rep. Author manuscript; Available in PMC 2026 Aug 12. (PMC13465167; doi:10.1016/j.celrep.2026.117507)
Supplement: Supplementary file 1 [file NIHMS2190748-supplement-1.pdf]

Cell Reports, Volume *45*

Supplemental information

Cell-type-specific proximity labeling of organ secretomes reveals energy balance-dependent proteomic remodeling

Kaja Plucińska, Charlotte R. Wayne, Henry Sanford, Boby Mathew, Nathalie Ropek, Stephanie M. Adaniya, Corey Model, Nicolás Gómez-Banoy, Ksenia Morozova, Xiongwen Cao, Jeffrey M. Friedman, Ken H. Loh, Paul Cohen, and Ekaterina V. Vinogradova

Supplementary figures.

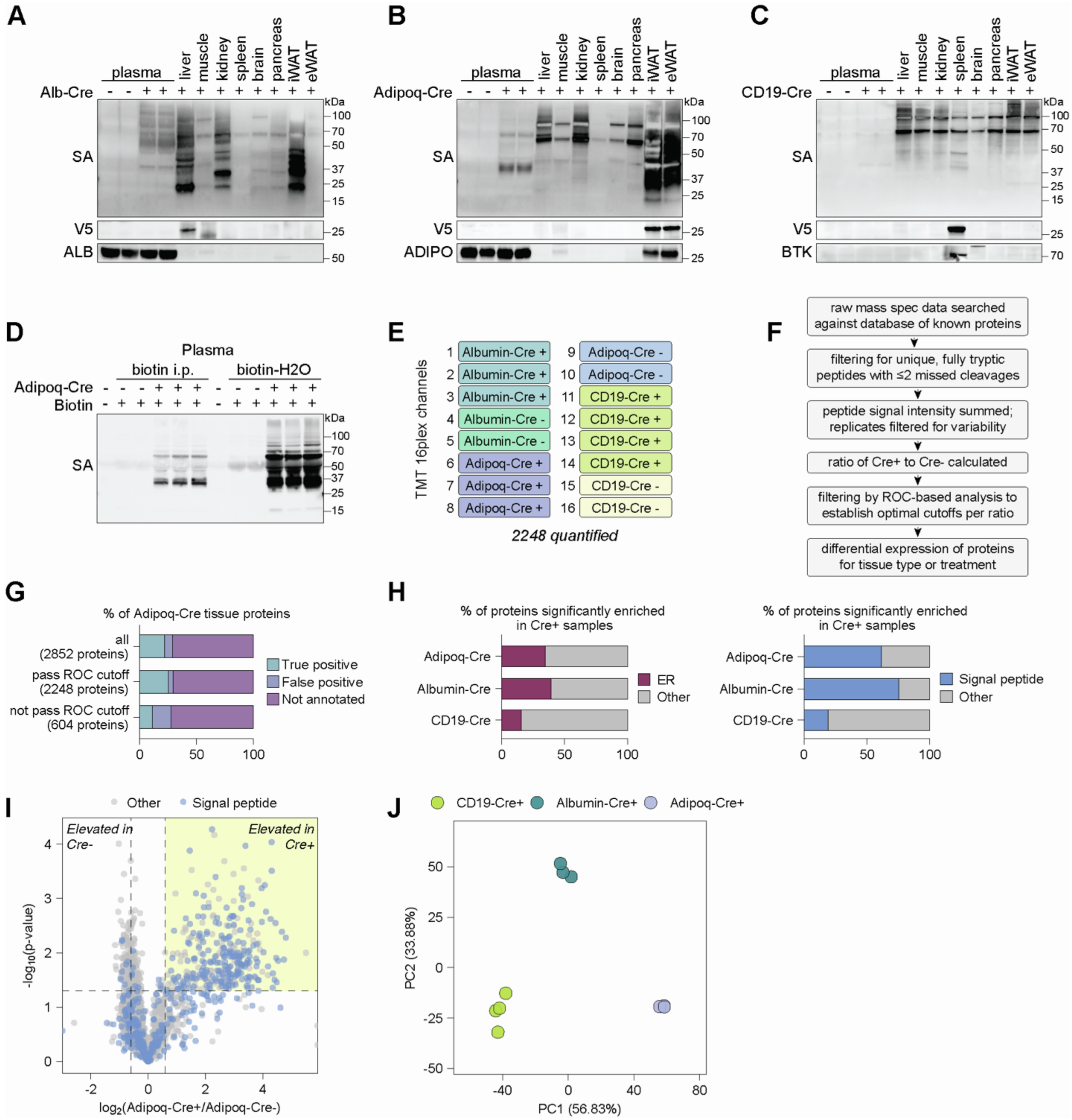

Figure S1. Characterization of a genetically encoded TurboID-KDEL in three tissue types, Related to Figure 1.

(A-C) Protein biotinylation and V5 expression in various tissue types and plasma assessed by Western blot analysis in (A) Albumin-TurboID^{KDEL}, (B) Adiponectin-TurboID^{KDEL}, and (C) CD19-TurboID^{KDEL} mice.

(D) Comparison of protein biotinylation efficiency in plasma from Adipoq-TurboID^{KDEL} mice treated with biotin (1 mg/mL) either intraperitoneally (i.p.) or in drinking water over the course of 7 days.

(E) Representative TMT channel assignments for a 16-plex LC-MS/MS/MS experiment comparing three tissue types under basal conditions. The number of proteins passing the ROC cutoff is also shown.

(F) General filters used for analysis of tissue mass spectrometry data.

(G) Representative bar plot showing annotation of proteins passing and failing ROC-based filtering in Adipoq-Cre samples. See supplementary methods for more details.

(H) Bar plot showing percentage of significantly enriched proteins (p-value < 0.05, FC > 1.5; corresponding to the highlighted region in I) annotated as secreted by SignalP (left) or as ER-associated by Gene Ontology (right).

(I) Representative volcano plot showing $\log_2$ fold changes of protein expression between Adipoq-Cre+ and Adipoq-Cre- samples. Dashed lines represent cutoffs of p-value < 0.05 and fold-change (FC) > 1.5. Signal peptides are labeled based on OutCyte predictions.

(J) Principal component analysis of proteomic data from Cre+ samples. Protein signal intensity values were $\log_2$ transformed before analysis.

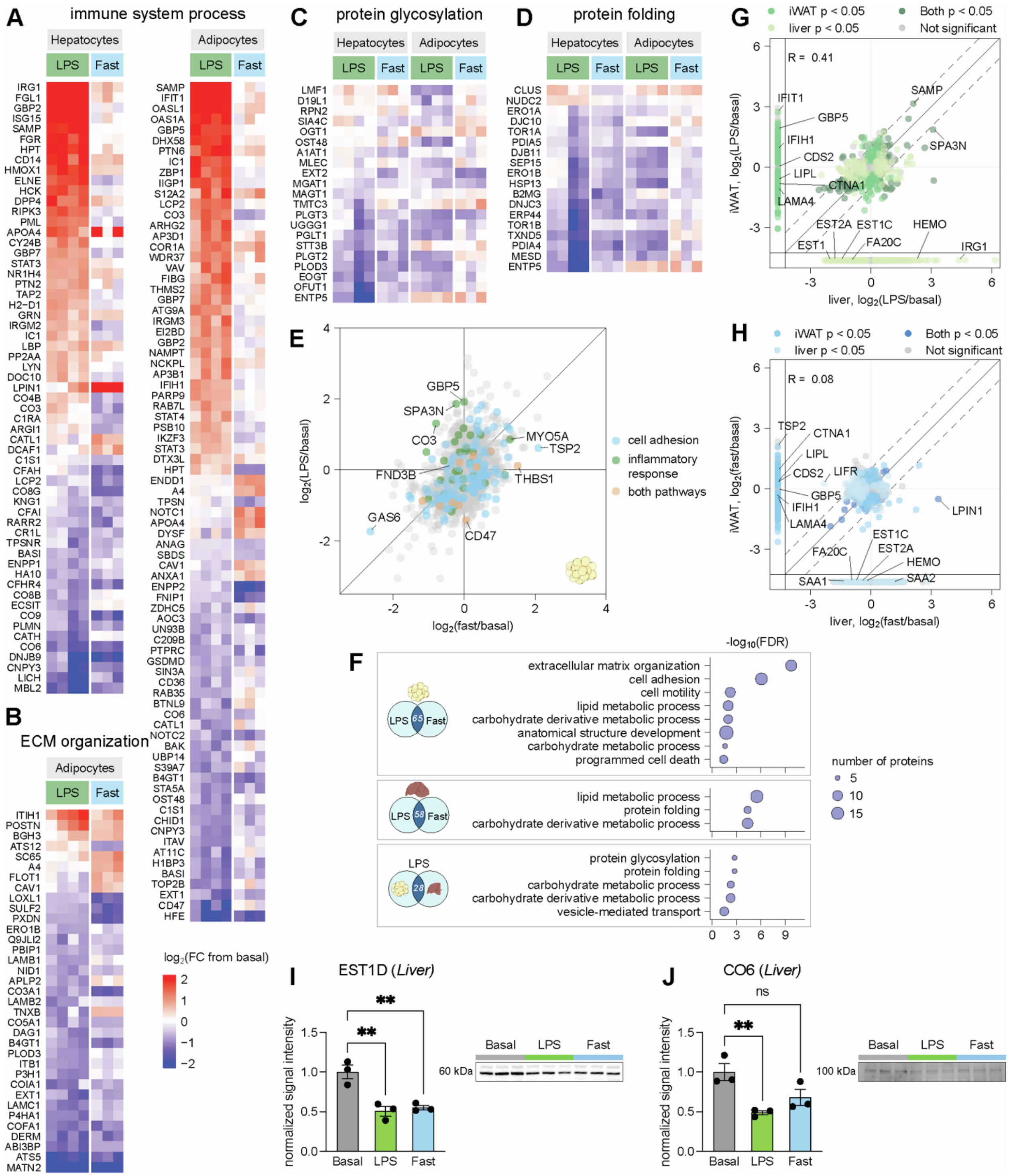

Figure S2. Comparison of proteomic responses to negative energy balance in hepatocytes and adipocytes, Related to Figure 2.

(A-D) Heat maps showing differentially expressed proteins contributing to (A) immune system process, (B) extracellular matrix (ECM) organization, (C) protein glycosylation, and (D) protein

folding GO-term enrichment in adipocytes or hepatocytes. Data is presented as $\log_2$(fold-change) compared to basal condition.

(E) Scatterplot showing correlation of $\log_2$(fold-change) values for protein expression changes in adipocytes from LPS-treated or fasted Adipoq-TurboID^{KDEL} mice relative to basal conditions. Proteins belonging to selected pathways are colored.

(F) Gene ontology (GO) analysis of pathways enriched at the intersection of proteins significantly downregulated in multiple conditions, including 65 proteins downregulated in adipocytes following LPS and fasting, 58 downregulated in hepatocytes in LPS and fasting, and 28 downregulated in both adipocytes and hepatocytes following LPS.

(G-H) Scatterplots of $\log_2$(fold-change) values for protein expression changes in liver (x) or iWAT (y) following LPS treatment (G) or fasting (H) compared to basal condition. Proteins quantified only in one cell type (derived from liver or iWAT) are shown on margins.

(I-J) Validation by Western blot of EST1D (I) and CO6 (J) downregulation in response to LPS and fasting in liver. Western blot band intensity was normalized to the total intensity of the corresponding lane in a stain-free gel image. Comparison performed by one-way ANOVA with Dunnett's multiple comparisons test (ns, $p > 0.05$; **, $p < 0.01$).

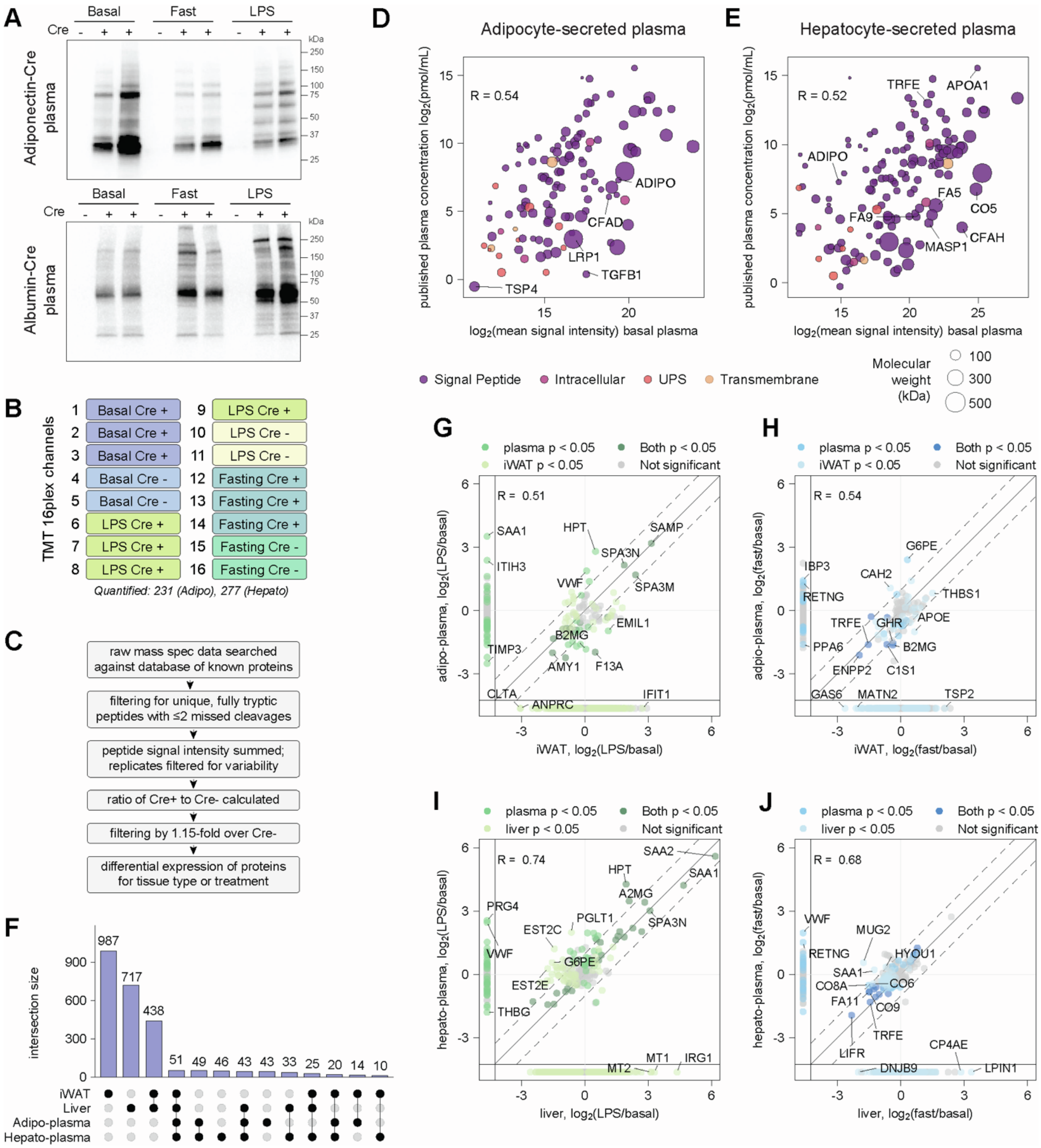

Figure S3. Hepato-plasma and adipo-plasma proteome responses to fasting or inflammation-induced anorexia, Related to Figure 3.

(A) Western blot showing TurboID-catalyzed biotinylation of proteins identified in plasma from biotin-treated Adipoq- and Albumin-TurboID^{KDEL} mice under basal and negative energy balance (fasting, LPS) conditions.

(B) Representative TMT channel assignment for a 16-plex LC-MS/MS/MS experiment comparing plasma proteomes from three conditions (basal, LPS, and fasting) in mice with and without a cell-type-specific Cre driver. The number of proteins passing the enrichment cutoff is also shown.

(C) Filters used for analysis of plasma mass spectrometry data.

(D-E) Scatterplots of log_2-transformed mean signal intensity values (x) for proteins quantified in basal adipo-plasma (D) and hepato-plasma (E) versus mean published absolute plasma concentrations (y). Size of points represents molecular weight in kilodaltons. Colors show SignalP and Outcyte secretion predictions.

(F) Upset plot of overlap in protein quantifications by TurboID-TMT between adipocytes, hepatocytes and plasma. Intersections with fewer than 10 proteins are not shown.

(G-J) Scatterplots of log_2(fold-change) values for expression changes in adipocyte (F-G) or hepatocyte (H-I) secreted proteins following LPS treatment (left) or fasting (right) compared to basal condition in plasma (x) and tissue-derived (y) samples. Proteins quantified in one sample (tissue or plasma) are shown on margins. R represents Pearson's correlation coefficient.

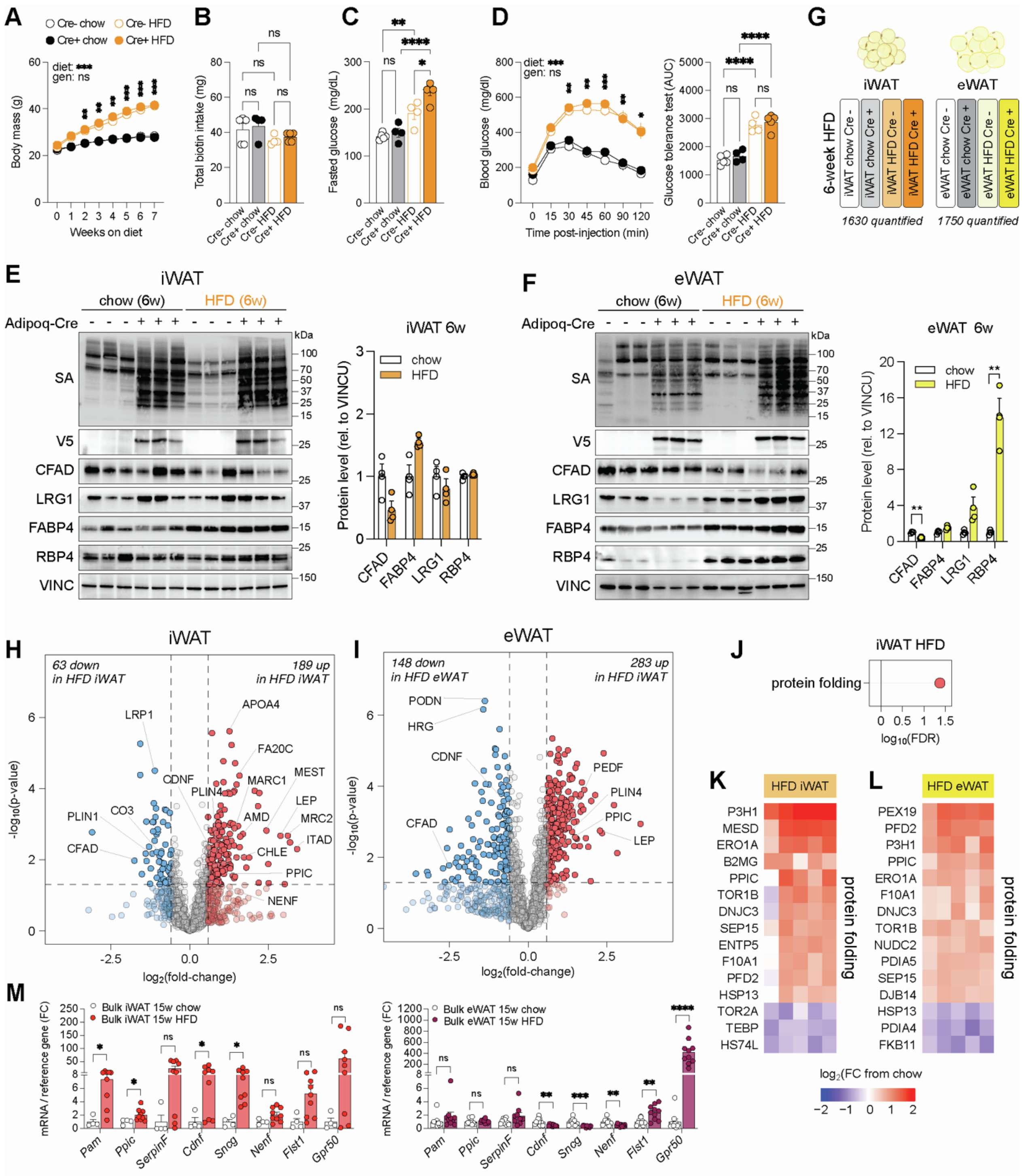

Figure S4. Proteomic responses of inguinal and epidydimal adipocytes in Adipoq-TurboID^{KDEL} mice to early obesity, Related to Figure 4.

(A) Body weights of Adipoq-TurboID^{KDEL} mice fed HFD or chow for 6 weeks. Biotin (1.5 mg/mL) was administrated in drinking water for 7 days on the last week of feeding. Conditions were

compared to basal by two-way ANOVA with Tukey's multiple comparisons test (ns, $p > 0.05$, not shown; *, $p < 0.05$; **, $p < 0.01$). Error bars show mean ± SEM; n = 4-6 mice per condition.

(B) Total biotin intake in mice after 7 days. Conditions were compared to basal using ordinary one-way ANOVA with Šidák multiple comparisons test (ns, $p > 0.05$). Error bars show mean ± SEM; n = 4-7 mice per condition.

(C) Fasted glucose in mice fed chow or HFD for 6 weeks. Conditions were compared to basal using ordinary one-way ANOVA with Šidák multiple comparisons test (ns, $p > 0.05$; *, $p < 0.05$; **, $p < 0.01$; ****, $p < 0.0001$). Error bars show mean ± SEM; n = 4-7 mice per condition.

(D) Glucose tolerance in mice fed chow or HFD for 6 weeks and area under curve (AUC). Conditions were compared to basal by two-way ANOVA with Tukey's multiple comparisons test (ns, $p > 0.05$, not shown; *, $p < 0.05$; **, $p < 0.01$; ***, $p < 0.001$). Error bars show mean ± SEM; n = 4-7 mice per condition. AUC was compared using ordinary one-way ANOVA with Šidák multiple comparisons test (ns, $p > 0.05$; ****, $p < 0.0001$). Error bars show mean ± SEM; n = 4-7 mice per condition.

(E-F) Western blot validation of protein biotinylation using streptavidin-HRP, tissue-specific V5 expression, and levels of known adipokines, including CFAD, LRG1, FABP4, and RBP4 with quantification in bulk iWAT (E) and eWAT (F) from Adipoq-TurboID^{KDEL} mice after 6 weeks of HFD feeding. Vinculin (VINC) was used as a loading control. Error bars show mean ± SEM, statistical comparisons by unpaired t-test (*, $p < 0.05$; **, $p < 0.01$).

(G) Representative TMT channel assignment for a 16-plex LC-MS/MS/MS experiment comparing proteomic changes induced by 6-week HFD vs. chow in iWAT or eWAT adipocytes from Adipoq-TurboID^{KDEL} Cre+ and Cre- mice (technical replicates not shown). The number of proteins passing the ROC cutoff in each experiment is also shown.

(H-I) Volcano plots showing $\log_2$(fold-changes) in secretory pathway protein expression in iWAT (H) and eWAT (I) samples following HFD feeding for 6 weeks relative to basal. Dashed lines represent cutoffs of p-value < 0.05 and fold-change > 1.5.

(J) Biological process over-represented in gene set enrichment affected by HFD feeding in iWAT-derived adipocytes at 6 weeks of HFD.

(K-L) Heat maps showing differentially expressed proteins contributing to protein folding GO-term enrichment in iWAT (K) and eWAT (L) samples following HFD feeding for 6 weeks. Data is presented as $\log_2$(fold-change) compared to the chow condition.

(M) RNA levels of HFD-regulated proteins in iWAT and eWAT from Adipoq-TurboID^{KDEL} mice after 15 weeks of HFD feeding. Conditions were compared to chow by t-test (ns, $p > 0.05$; *, $p < 0.05$;

**, $p < 0.01$; ***, $p < 0.001$; ****, $p < 0.0001$). Error bars show mean ± SEM; n = 4-7 mice per condition.

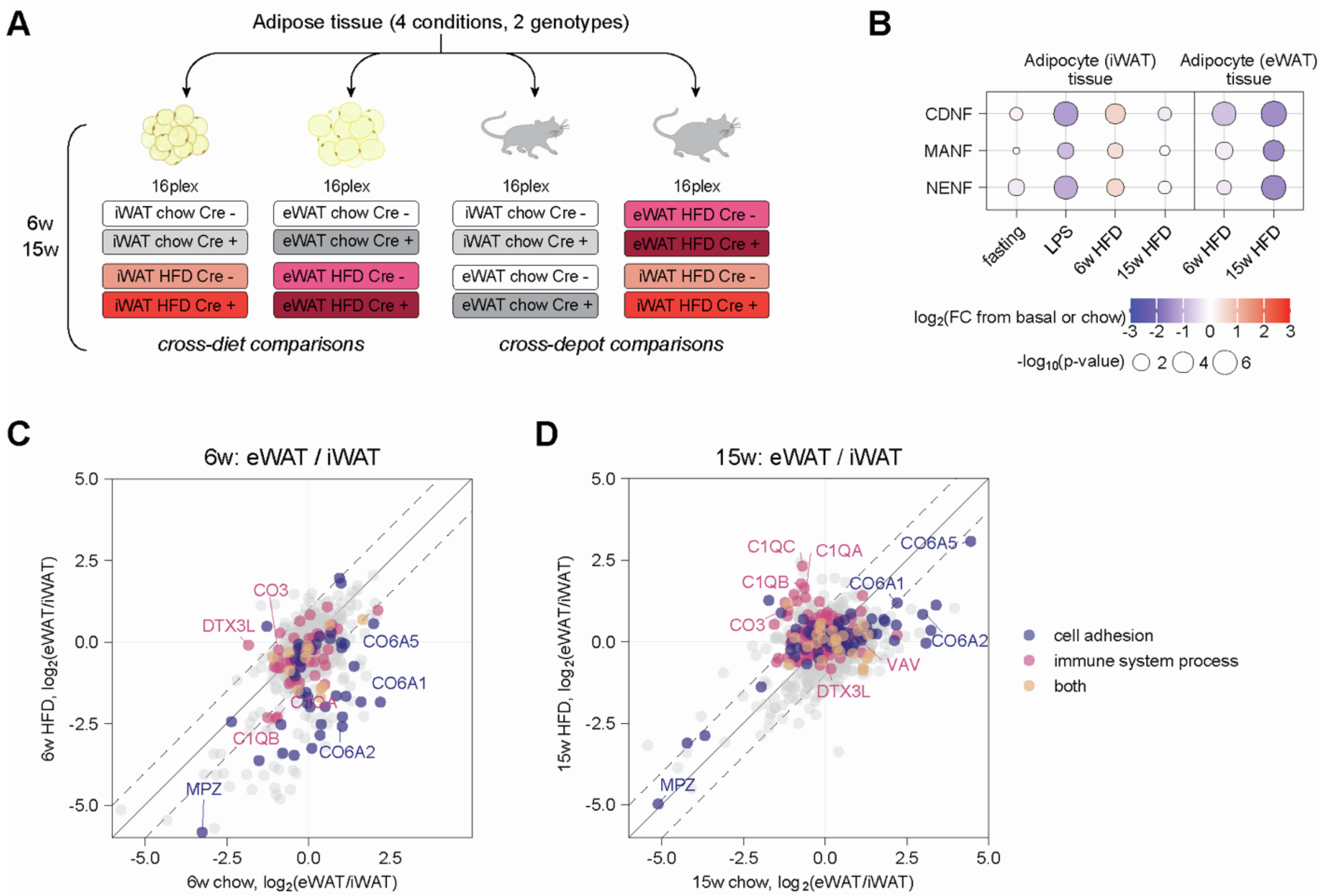

Figure S5. Depot-specific protein remodeling in inguinal and epididymal adipocytes in early and advanced obesity, Related to Figure 5.

(A) Schematic showing TurboID-TMT MS experiments for cross-diet and cross-depot comparisons (technical replicates not shown).

(B) Normalized mean signal intensity values for CDNF, MANF, and NENF proteins across negative energy balance and high-fat diet experiments and depot.

(C-D) Correlational analysis of log_2(fold-changes) in protein expression following chow and HFD feeding for 6 weeks (C) and 15 weeks (D) in inguinal compared to epididymal adipocytes. Proteins belonging to selected pathways are colored.

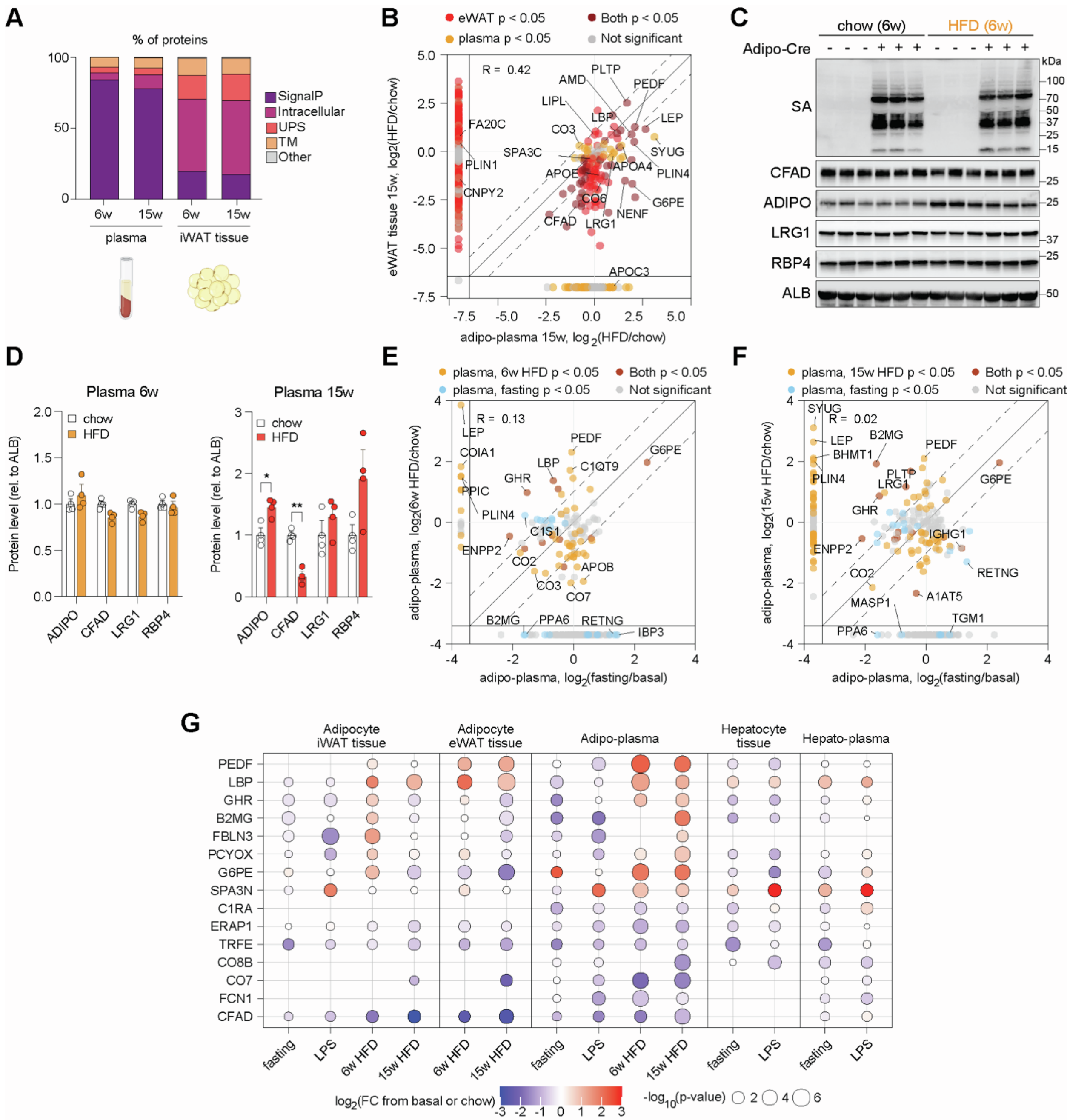

Figure S6. Characterization of adipo-plasma proteome during obesity, Related to Figure 6.

(A) Bar plot of proteins quantified in Adipoq-TurboID^{KDEL} iWAT-derived adipocytes and adipo-plasma proteomes at 6 and 15 weeks of DIO, with protein class labeled based on SignalP and OutCyte predictions.

(B) Scatterplot of log_2(fold-changes) in protein expression in eWAT vs. plasma proteomes of Adipoq-TurboID^{KDEL} mice following HFD feeding for 15 weeks compared to chow. Proteins quantified for only one of the conditions are shown on margins.

(C) Western blot validation of protein biotinylation using streptavidin-HRP and levels of known adipokines, including CFAD, ADIPO, LRG1, and RBP4 in bulk plasma samples from Adipoq-TurboID^{KDEL} mice after 6 weeks of HFD feeding. Albumin (ALB) was used as a loading control.

(D) Quantification of protein levels in bulk plasma from Adipoq-TurboID mice fed chow or HFD. Error bars show mean ± SEM, statistical comparisons by unpaired t-test (*, $p < 0.05$; **, $p < 0.01$).

(E-F) Scatterplots of $\log_2$(fold-changes) in protein expression in plasma proteomes of Adipoq-TurboID^{KDEL} mice following 48-hour fasting and HFD feeding for 6 weeks (E) or 15 weeks (F) compared to basal or chow. Proteins quantified for only one of the conditions are shown on margins.

(G) Dot plot of $\log_2$(fold-changes) and p-values of selected proteins detected in adipocytes, hepatocytes, and plasma across negative and positive energy balance conditions. Data are shown relative to own control group (e.g. fasting vs. basal, LPS vs. basal, HFD vs. chow).

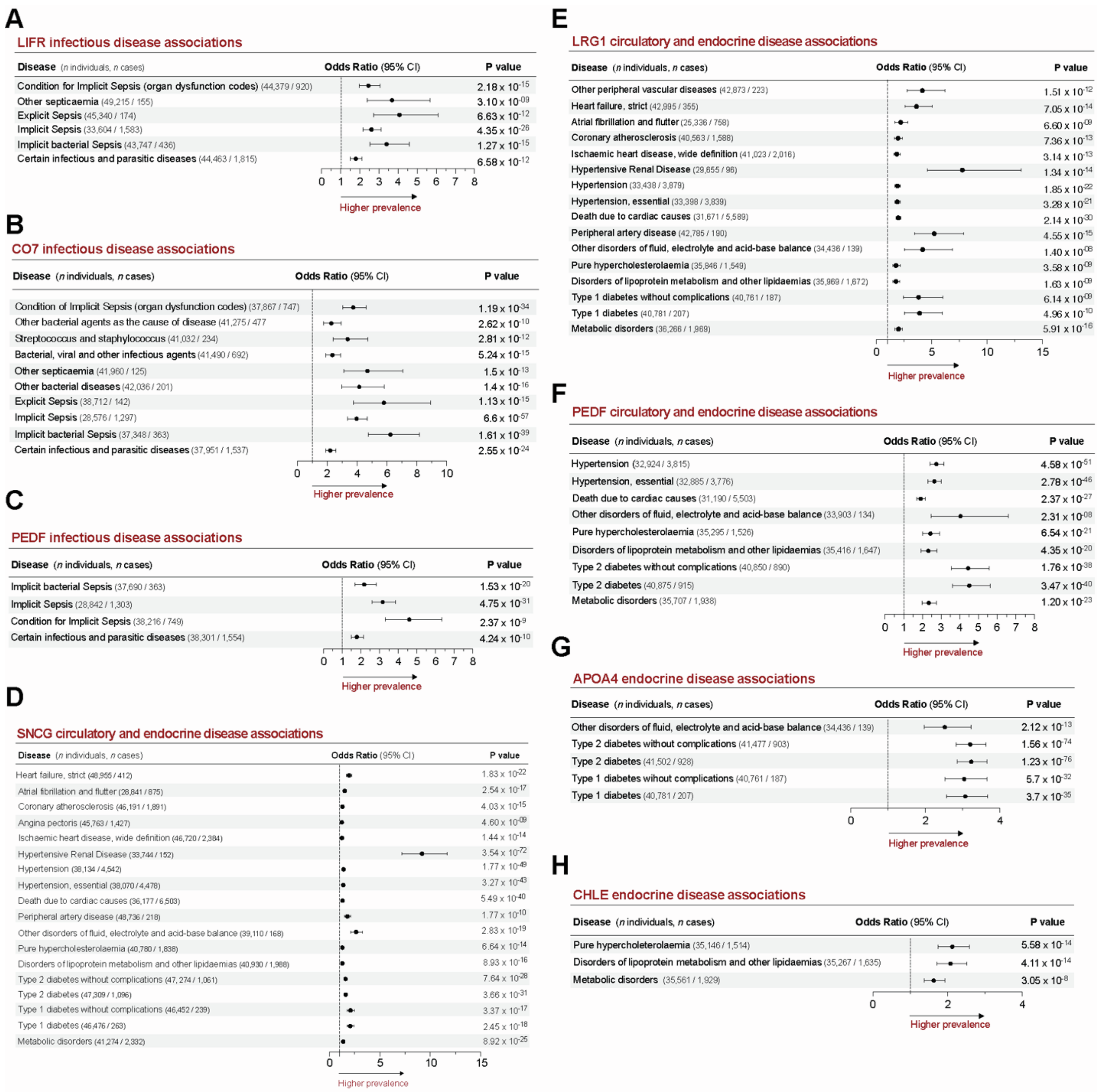

Figure S7. Odd ratios for circulatory, endocrine, and infectious disease and clinical outcomes linked with individual circulating proteins derived from adipocytes or hepatocytes in various energy balance states, Related to Figure 7.

(A-H) Odds ratios for infectious diseases, endocrine, and/or circulatory prevalence-based outcomes linked with basal circulating levels of leukemia inhibitory factor receptor (LIFR), complement component 7 (CO7), pigment epithelium-derived factor (PEDF), gamma synuclein (SNCG), leucine-rich glycoprotein 1 (LRG1), apolipoprotein A-IV (APOA4), and cholinesterase (CHLE) in Health and Disease Atlas based on UKBB cohort of 53,026 individuals.
